# Utility of whole exome sequencing analysis in differentiating intrapulmonary metastatic multiple ground-glass nodules (GGNs) from multiple primary GGNs

**DOI:** 10.1007/s10147-022-02134-8

**Published:** 2022-02-16

**Authors:** Dong Zhou, Quan-Xing Liu, Man-yuan Li, Bin Hou, Gui-xue Yang, Xiao Lu, Hong Zheng, Li Jiang, Ji-Gang Dai

**Affiliations:** Department of Thoracic Surgery, Xinqiao Hospital, Third Military Medical University (Army Medical University), Chongqing, 400037 China

**Keywords:** Lung cancer, Multiple ground-glass nodule (GGN), Whole exome sequencing (WES), Clone evolution analysis

## Abstract

**Purpose:**

Clinical evidence of metastasis with ground-glass nodules (GGNs) has been reported, including pulmonary metastasis and distant metastasis. However, the clonal relationships of multiple GGNs at the genetic level remain unclear.

**Experimental design:**

Sixty tissue specimens were obtained from 19 patients with multiple GGN lung cancer who underwent surgery in 2019. Whole exome sequencing (WES) was performed on tissue samples, and genomic profiling and clone evolution analysis were conducted to investigate the genetic characteristics and clonality of multiple GGNs.

**Results:**

A total of 15,435 nonsynonymous mutations were identified by WES, and GGNs with shared nonsynonymous mutations were observed in seven patients. Copy number variant (CNV) analysis showed that GGNs in ten patients had at least one shared arm-level CNV. Mutational spectrum analysis showed that GGNs in three patients had similar six substitution profiles and GGNs in fou patients had similar 96 substitution profiles. According to the clone evolution analysis, we found that GGNs in five patients had shared clonal driver gene mutations. Taken together, we identified that 5 patients may have multiple primary GGNs without any similar genetic features, 2 patients may have intrapulmonary metastatic GGNs with ≥ 3 similar genetic features, and the other 12 patients cannot be determined due to insufficient evidences in our cohort.

**Conclusions:**

Our findings suggest that the intrapulmonary metastasis exist in multiple GGNs, but the number of GGNs was not associated with the probability of metastasis. Application of genomic profiling may prove to be important to precise management of patients with multiple GGNs.

**Supplementary Information:**

The online version contains supplementary material available at 10.1007/s10147-022-02134-8.

## Introduction

With the widespread use of low-dose chest computed tomography (CT) and lung cancer screening, more patients with early-stage lung cancer were found, including ground-glass nodules (GGNs). The incidence of pulmonary GGNs has been reported to be more than 60% [1, 2], and up to 10% of them harbor multiple GGNs [3]. GGNs are traditionally considered as multiple primary lung cancers and at the early stage of tumorigenesis [4–6]. However, to date there is still no consensus regarding the optimal management of patients with multiple pulmonary GGNs, mainly due to the difficulty of determining whether multiple GGNs in a patient indicate intrapulmonary metastasis or multifocal origin. Two major mechanisms have been proposed for histologically similar multifocal tumors: i, a single clonal event resulting in a tumor that subsequently spreads within one or both lungs (intrapulmonary metastasis); ii, multiple tumors arising independently in a carcinogen-damaged field (field cancerization, multiclonality) [7].

Clinical evidence of metastasis with GGNs has been reported, including pulmonary metastasis [8, 9] and distant metastasis [10–14]. At present, a few studies have been focused on whether multiple GGNs were multiple primaries or intrapulmonary metastasis [15–18], most of which were based on the targeted genes [15–17], and only one study was based on genomics [18]. The targeted gene based studies did not find any evidence of intrapulmonary metastasis. The genomics based study was the first report of GGNs with intrapulmonary metastasis at the genetic level, however, only two patients were included.

To more accurately elucidate the clonality of multiple GGNs, we performed whole exome sequencing (WES) on 60 GGNs from 19 patients. Copy number variation (CNV) profiles, somatic mutations, six substitution profiles, and 96 substitution profiles were compared between multiple GGNs, and clone evolution analysis was used to investigate their clonal relationships.

## Material and methods

### Patients and samples

Sixty tissue specimens of GGNs were acquired from 19 treatment-naïve patients with lung cancer who underwent surgery at Xinqiao Hospital between May and November 2019. This study protocol was approved by the ethics committee of the Army Medical University, Chongqing, China and informed consent was obtained from all the patients before the study initiation.

### Whole exome sequencing

We extracted genomic DNA from formalin-fixed paraffin-embedded pulmonary tumor tissue samples using Tissue Kit (69504, QIAGEN, Venlo, the Netherlands) according to the manufacturer’s instructions. The tissue sections were examined by two pathologists (ZY, XP) independently and were required to contain at least 50% tumor cell nuclei with less than 20% necrosis per TCGA protocol requirements. The DNA was isolated by targeted capture pulldown and exon-wide libraries were generated from native genomic DNA using the xGen® Exome Research Panel (Integrated DNA Technologies, Inc., Skokie, Illinois, US) and TruePrep DNA Library Prep Kit V2 for Illumina (#TD501, Vazyme, Nanjing, China) according to the manufacturers’ instructions. Paired-end sequence data were generated using Illumina HiSeq machines with an average sequencing depth of 168.4 × for normal tissues and 246.2 × for tumor tissues. The sequence data were aligned to the human reference genome (NCBI build 37) using Burrows–Wheeler Aligner (BWA), and polymerase chain reaction (PCR) duplicates were sorted and removed using sambamba.

### Variant calling pipeline

Single nucleotide variants (SNVs), insertions, and deletions were detected using Strelka2 with default parameters. Variants and polymorphisms were annotated using the Ensembl Variant Effect Predictor. A minimum of 20 reads covering mutated region and 5 reads supporting the variant allele were required for somatic SNV/indel calling. In contrast, sequencing depth need to be ≥ 20x, and reads supporting the variant < 5 at the same site in the normal control sample. Variants with minor allele frequency (MAF) > 1% in the ExAC, gnomAD, and esp6500 databases were filtered out as common germline variants.

### Copy number variant analysis

Somatic copy number variations (CNVs) were analyzed using FACETS, and the resulting CNVs were used in further analysis.

### Mutational signature

The mutational signatures, defined by the triplets of nucleotides around each mutation of each sample, were deconvoluted into mutational processes using MutationalPatterens.

### Clone evolution analysis

PyClone [1] was used to cluster subclones inferred from SNVs. With the clustering results used as input, the optimal tree solutions were obtained with the iterative version of citup. The cancer cell fraction (CCF) of somatic SNVs in primary and metastatic pairs was calculated using PyClone and was estimated as a surrogate of tumor clonal architecture. The CCF of a mutation indicates the proportion of cells in the tumor sample that harbor that mutation. Theoretically, mutations with similar CCF values tend to occupy the same proportion of cancer cells and cluster together in the CCF plots, indicating the existence of a cancer cell clone. This clustering is performed using Bayesian clustering that jointly estimates the CCF values and number of populations based upon the set of CCF distributions from each sample. Two-dimensional density plots showed the CCF distribution of pairwise samples. In each sample, a particular mutation was considered as subclonal if the defined clone comprising this mutation had a mean CCF value less than 0.8.

### Statistical analysis

Pearson’s correlation analysis was carried out to determine the correlation between 96 substitution profiles of tumors. *T* test was used to determine the difference between six substitution profiles.

## Results

### Patient characteristics

Sixty GGNs were acquired from 19 patients with lung cancer (median age, 48.0 years; 5/19 males), including 11 patients with 2 GGNs, 4 patients with 3 GGNs, 1 patient with 4 GGNs, 2 patients with 5 GGNs, and 1 patient with 12 GGNs. The clinicopathologic characteristics of 19 patients are shown in Table S1.

### Copy number variations

The profiles of copy number variations (CNVs) for each of 19 cases are shown in Fig. S1. No arm-level CNVs were detected in one patient (P03) (Fig. S1A). Eight patients had different arm-level CNV profiles, of which 7 patients (P01, P02, P04, P05, P06, P10 and P13) harbored arm-level CNVs in only one GGN, while no CNVs were detected in the other GGNs. Both GGNS in Patient 11 had arm-level CNVS, but they were different (Fig. S1B). Ten patients shared at least one arm-level CNV between GGNs from an individual patient (Fig. S1C). There were o shared arm-level CNV in two patients (P12, P17), 2 shared arm-level CNVs in four patients (P07, P08, P14, P16), 3 shared arm-level CNVs in one patient (P09), 4 shared arm-level CNVs in one patient (P18), 7 shared arm-level CNVs in one patient (P15), and 19 shared arm-level CNVs in one patient (P19).

### Somatic mutations

**Fig. 1:**
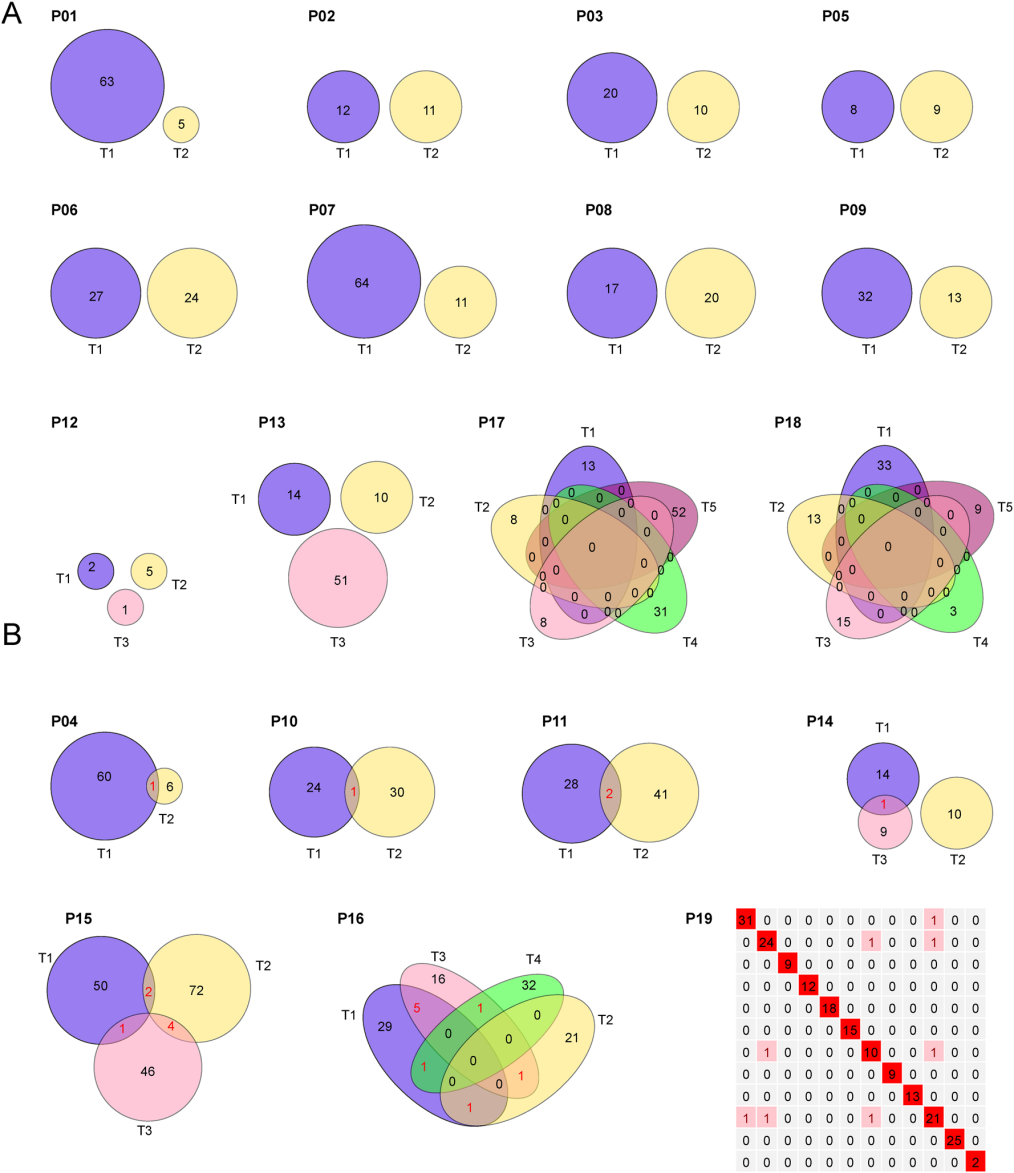
Venn diagrams of nonsynonymous somatic mutations for multiple ground-glass nodules (GGNs) in each patient. **A** Patients without shared nonsynonymous somatic mutations in different GGNs. **B** Patients harboring shared nonsynonymous somatic mutations in different GGNs

A total of 18575 mutations (15435 nonsynonymous) in 8733 genes (7876 nonsynonymous) were identified, including 18548 SNVs (15408 nonsynonymous) and 27 indels. Mutated cancer-related genes in at least two samples are presented in Fig. S2A. Twelve of 19 patients did not share any nonsynonymous mutations (Fig. 1A). Only one nonsynonymous mutation was found to be shared between GGNs in three patients (P04, P10, P14), two shared nonsynonymous mutations were found in two patients (P11, P19), and more than four shared nonsynonymous mutations were found in two patients (P15, P16) (Fig. 1B, Fig. S2B). Among the seven patients who harbored shared nonsynonymous mutations between GGNs in an individual patient, five patients (P11, P14, P15, P16 and P19) harbored shared cancer-related mutations (Fig. S2A).

### Mutational spectrum

**Fig. 2:**
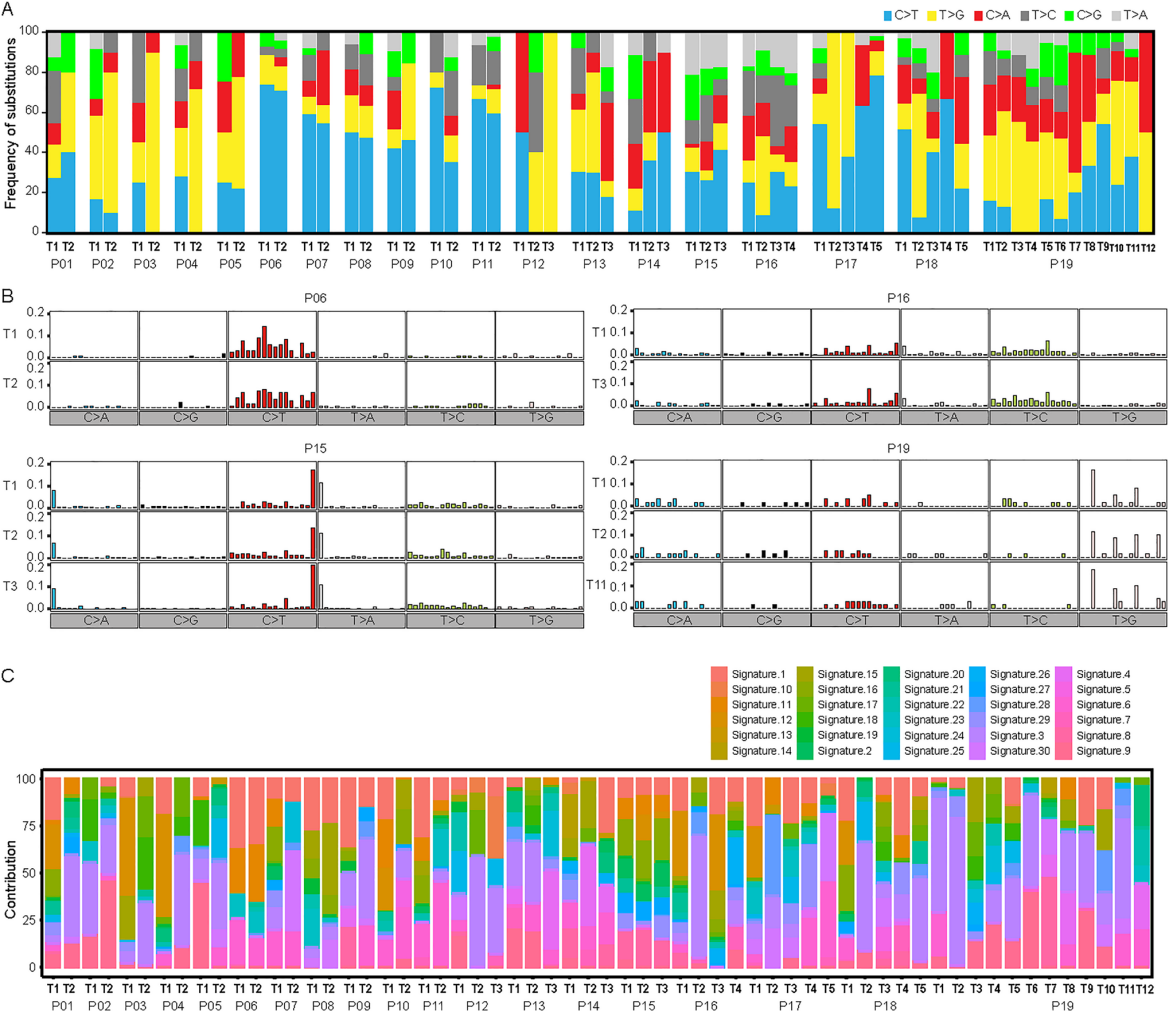
Mutational spectrum analysis for multiple ground-glass nodules (GGNs) in each patient. **A** Bar plots showing the frequency of six substitutions. **B** Bar plots showing the frequency of 96 substitutions. **C** Bar plots showing the contribution of 30 COSMIC mutational signatures

Mutational spectrum of six substitutions showed that C > T transition or T > G transversion was dominant in most GGNs except for three GGNs with dominant C > A transversion (T3 of P13, T5 of P18, T7 of P19) (Fig. 2A). The median percentages of variants of C > T, T > G, C > A, T > C, C > G, and T > A were 30.2%, 17.4%, 13.3%, 9.1%, 6.2%, and 2.8%, respectively. Significantly discordant mutational spectra were observed between different GGNs in most patients at the level of substitution composition (*t* test, *p* < 0.05) except four pairs of tumors in three patients. As shown in Fig. S3, T1 and T2 of P06, T1 and T4 of P16, T1 and T5 of P19, and T5 and T6 of P19 had similar six substitution profiles (*t* test, *p* > 0.05).

The profiles of 96 substitutions for each GGN are presented in Figure S4. The correlation coefficients between GGNs calculated by 96 substitution profiles are listed in Table S2. For most patients, 96 substitution profiles varied between GGNs from an individual patient, while GGNs from four patients (P6, P15, P16 and P19) had similar 96 substitution profiles (Pearson coefficient > 0.8; Fig. 2B). Among 30 COSMIC mutational signatures, signature 1, 3 and 6 had high proportion (median 9.6%, 10.9% and 2.4%, respectively) in this cohort (Fig. 2C).

### Clone evolution analysis

We used high-confidence somatic mutations to investigate the clonal relationship between different GGNs from each patient. Two-dimensional plotting of cancer cell fractions (CCF), the fraction of cancer cells that harbor each somatic mutation, showed the distribution of clusters (Fig. S5). According to the CCFs of clusters, the patients were categorized into two groups including 14 patients without shared clonal driver mutations (Fig. S5A) and 5 patients with shared clonal driver mutations (Fig. S5B). As shown in Fig. S5A, different GGNs from an individual patient did not share clonal driver mutations, indicating that they were probably independent primaries. Among the patients with shared clonal driver mutations (Fig. S5B), different GGNs from P15 or P16 also shared multiple clones with passenger mutations, suggesting that they may have metastatic disease.

### GGN with multiple primaries or intrapulmonary metastasis

To identify the multiple GGNs as multiple primary tumors or intrapulmonary metastatic tumors, we combined the different results including CNV profile, shared somatic mutations, 6 substitution profiles, 96 substitution profiles and clone evolution diagram, and investigated the consistency between different results. The results are summarized in Table 1 and Table S3. If there are ≥ 3 similar genetic features between tumors from an individual patient, they are considered as multiple primary GGNs; if there are ≥ 3 similar genetic features, they are considered as the possibility of intrapulmonary metastasis; otherwise, they are considered ambiguous. According to this criterion, 5 patients (P01, P02, P03, P05 and P13) may be identified with multiple primary GGNs, 3 patients (P06, P15 and P16) may be identified with GGNs with intrapulmonary metastasis, and in 11 patients cannot be determined due to insufficient evidences. When taken together, however, it is worth noting that patient 06, while meeting our criteria of having three similar genetic characteristics between two nodules, had two substitution profiles and no shared somatic mutation or CNV profiles between them. This conclusion should be taken with caution.

**Table 1:** The consistency of genetic characteristics for each tumor pair in patient 1–18

Patient ID	Tumor pair	CNV profile	Shared somatic mutations	Six substitution profile	Ninety-six substitution profile	Clone evolution diagram
P01	T1 vs T2					
P02	T1 vs T2					
P03	T1 vs T2					
P04	T1 vs T2		√			√
P05	T1 vs T2					
P06	T1 vs T2			√	√	√
P07	T1 vs T2	√				
P08	T1 vs T2	√				
P09	T1 vs T2	√				
P10	T1 vs T2		√			
P11	T1 vs T2		√			√
P12	T1 vs T2					
	T1 vs T3	√				
	T2 vs T3					
P13	T1 vs T2					
	T1 vs T3					
	T2 vs T3					
P14	T1 vs T2					
	T1 vs T3	√	√			
	T2 vs T3					
P15	T1 vs T2	√	√		√	√
	T1 vs T3	√	√		√	√
	T2 vs T3		√		√	√
P16	T1 vs T2		√			√
	T1 vs T3	√	√		√	√
	T1 vs T4		√	√		√
	T2 vs T3		√			√
	T2 vs T4	√				√
	T3 vs T4		√			√
P17	T1 vs T2					
	T1 vs T3					
	T1 vs T4					
	T1 vs T5					
	T2 vs T3					
	T2 vs T4					
	T2 vs T5					
	T3 vs T4					
	T3 vs T5					
	T4 vs T5	√				
P18	T1 vs T2	√				
	T1 vs T3	√				
	T1 vs T4	√				
	T1 vs T5	√				
	T2 vs T3					
	T2 vs T4					
	T2 vs T5	√				
	T3 vs T4					
	T3 vs T5	√				
	T4 vs T5	√				

The result for patient 19 iss shown in Table S3

“√” indicates that the genetic characteristics of pairwise samples are consistent or similar

**Fig. 3:**
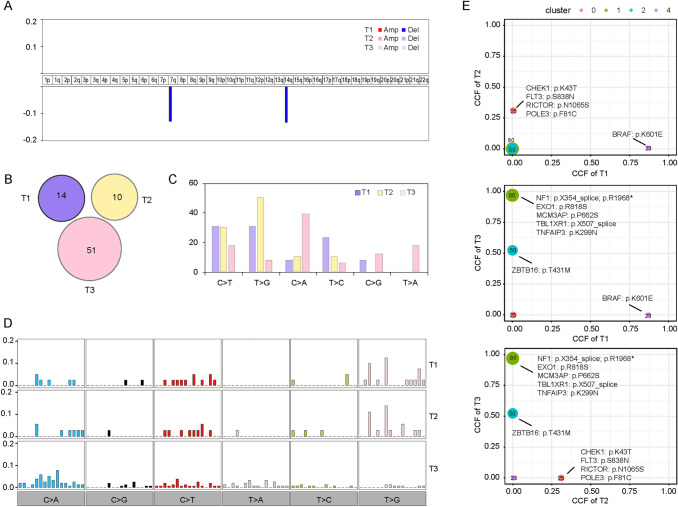
A typical case (P13) with multiple primary ground-glass nodules (GGNs). **A** Three GGNs did not share any chromosome arms with CNVs. **B** Three GGNs did not share any nonsynonymous mutations. **C** The profiles of six substitutions were significantly different in three GGNs. **D** The profiles of 96 substitutions were significantly different in three GGNs. **E** None of the clones or subclones were shared by three GGNs

A case (P13) with multiple primary GGNs is presented in Fig. 3. Three tumors did not share any chromosome arms with CNVs (Fig. 3A) or any nonsynonymous mutations (Fig. 3B). The profiles of 6 substitutions (Fig. 3C) and 96 substitutions (Fig. 3D) were significantly different in three tumors. In the two-dimensional diagrams (Fig. 3E), each spot represents a mutation cluster, and each cluster has multiple mutations. The coordinates of the spots in the figure represent the mean CCF of each mutation in each cluster, and the size of the spots represents the number of mutations in each cluster. Moreover, the driver gene mutations were also labeled in the two-dimensional diagrams, with spots on the X-axis or Y-axis representing private clonal driver gene mutations in a nodule, and spots near the diagonal representing clonal driver gene mutations shared by both nodules. None of the clones or subclones were shared by the three tumors (Fig. 3E). T1 had a private clone with 23 somatic mutations, T2 had a private subclone with 25 somatic mutations, and T3 had a private clone and a private subclone with 80 and 50 somatic mutations, respectively.

**Fig. 4:**
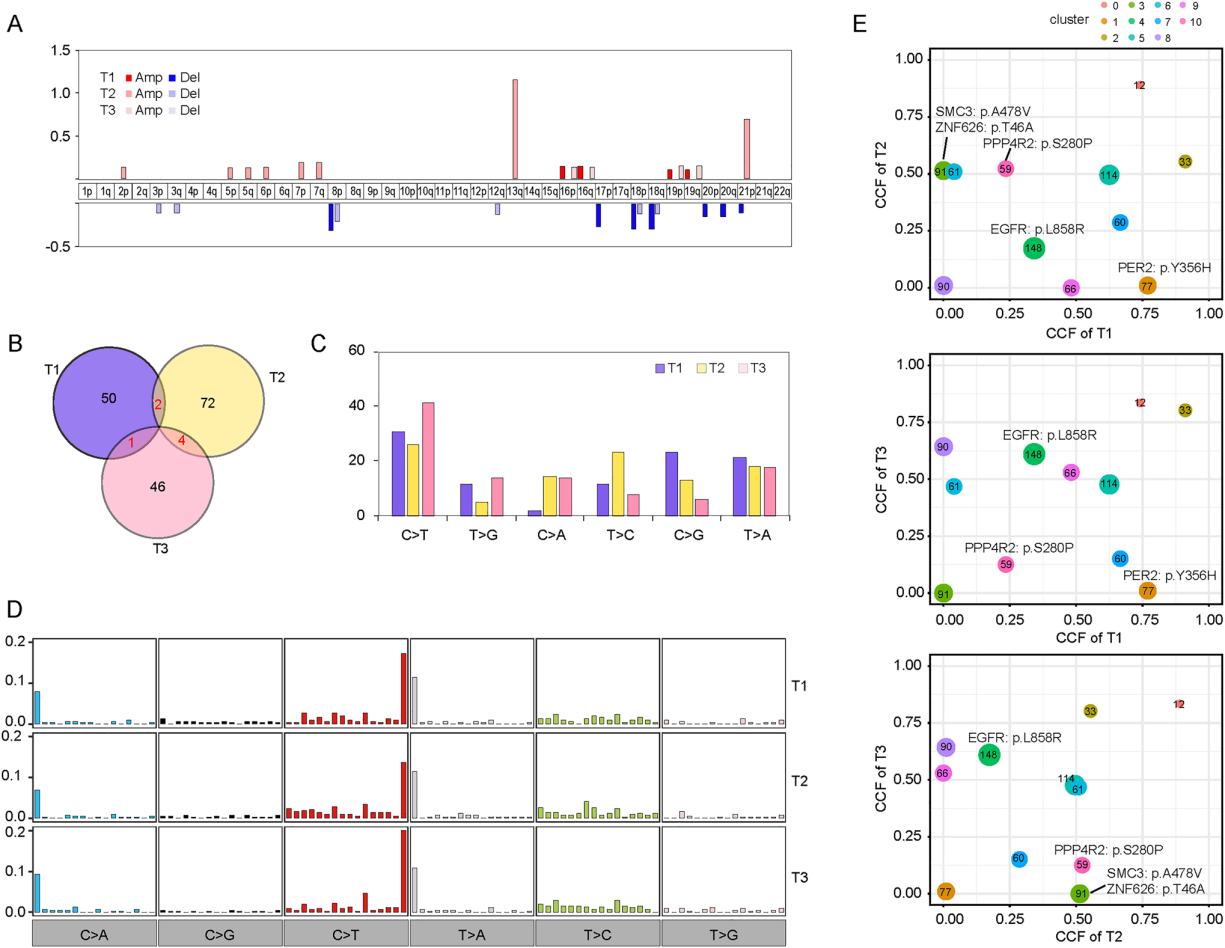
A typical case (P15) with intrapulmonary metastatic ground-glass nodules (GGNs). **A** Different GGNs had CNV amplifications at chromosome arms 16p, 16q, 19p and 19q, and had CNV deletions at chromosome arms 8p,18p and 18q. **B** Nonsynonymous mutations were shared by different GGNs. **C** Different GGNs had similar profiles of six substitutions. **D** Different GGNs had similar profiles of 96 substitutions. **E** Different GGNs shared clones and subclones

A case (P15) with intrapulmonary metastatic GGNs is presented in Fig. 4. As shown in Fig. 4A, both tumors had CNV amplifications at chromosome arms 16p, 16q, 19p and 19q, and had CNV deletions at chromosome arms 8p,18p and 18q. The common nonsynonymous mutations shared by two tumors accounted for about 1.0–3.1% of total nonsynonymous mutations in both tumors (Fig. 4B). The profiles of six substitutions (Fig. 4C) as well as 96 substitutions (Fig. 4D) of two tumors were similar. Clone evolution analysis showed a shared clone with 12 somatic mutations in all three tumors and a shared clone with 33 somatic mutations in T1 and T3. In addition, four subclones were shared in tumor pairs including driver gene mutations (Fig. 4E).

Of the other 11 patients with insufficient evidence for the identification of multiple primary or intrapulmonary metastatic GGNs, 6 patients (P07, P08, P09, P12, P17 and P18) merely had similar CNV profiles between different GGNs, while other genetic features were different. As shared arm-level CNV was commonly observed in this cohort (10/19), but it may not be evidence of intrapulmonary metastasis. Thus, these six patients were considered to have multiple primary GGNs. For P04, there was one nonsynonymous mutation (ZNF730) shared by two tumors (Fig. 1B, Fig. S2B). The clone evolution analysis showed that two tumors shared a cluster with 16 mutations, which was a clone in T1 but a subclone in T2 (Fig. S5B). However, the profiles of arm-level CNVs (Fig. S1B), six substitutions (Fig. 2A) or 96 substitutions (Fig. S4) were significantly different. Considering the shared cluster containing the mutation of EGFR that may be caused by parallel evolution, we considered tumors of P04 as two primary GGNs. For P10, only one nonsynonymous mutation (ZNF783) was shared by two tumors (Fig. 1B, Fig. S2B) and other genetic features were different, so we considered tumors of P10 as two primary GGNs. For P11, there were two nonsynonymous mutations (USP6 and PABPC1) shared by two tumors, both of which were cancer-related mutations (Fig. 1B, Fig. S2A). The clone evolution analysis showed that two tumors shared a clone with six mutations including PABPC1 (Fig. S5B). Moreover, although two tumors had similar 96 substitution profiles (Fig. S4), the correlation coefficient was < 0.8 (that is, 0.71) (Table S2). The conclusions regarding intrapulmonary metastasis GGNs should therefore be taken with caution. For P14, two arm-level CNVs and one nonsynonymous mutation (ERBB2) were shared by two tumors; nevertheless, the clone evolution diagram showed they were completely independent. We considered GGNs of P14 as multiple primary tumors. For P19 with 12 GGNs, the clone evolution diagram showed no shared clonal driver mutations between tumors (Fig. S5A). A nonsynonymous mutation (CACNA1I) was shared by T1 and T10, and a driver mutation (KRAS) was shared by T2, T7 and T10. The profiles of six substitutions were similar between T1 and T5, as well as T5 and T6 and the profiles of 96 substitutions were similar between T1 and T11, as well as T2 and T11. However, besides the CNV profile, only one genetic feature was similar for each tumor pair. Thus, we considered tumors of P19 as multiple primary GGNs.

Finally, we identified that 5 patients (P01, P02, P03, P05 and P13) may have multiple primary GGNs, 2 patients (P15 and P16) may have intrapulmonary metastatic GGNs, and the other 12 patients cannot be determined due to insufficient evidences in our cohort.

## Discussion

In this study, we collected 60 GGNs from 19 patients with lung cancer to explore the evolutionary relationship between GGNs in these patients. Histological examination revealed that 78.3% (47/60) of GGNs were minimally invasive adenocarcinoma (MIA) and 18.3% (11/60) were adenocarcinoma in situ (AIS) (Table S1). It seems difficult to determine their relationship between GGNs only from the pathological morphology. For example, patients P02, P03, P05, P18, P19, etc. were similar in morphology, but differed greatly in genetic characteristics, emphasizing the significance of molecular technology for correct clinical diagnosis. We performed WES analysis on 19 patients with multiple GGNs, and as a result, patients with multiple primary GGNs accounted for 26.3% (5/19) and patients with intrapulmonary metastatic GGNs accounted for 10.5% (2/19). This is the first study to introduce clone evolution analysis to study multiple GGNs, which is a reliable molecular method to explore the clonal relationships between tumors. Molecular analysis has been used to study whether multiple GGNs are multiple primaries or intrapulmonary metastasis in the previous studies [15–18]. As far as we know, Chung et al. were the first to use molecular analysis to study multiple GGNs [15]. They analyzed mutational status of the *EGFR* and *KRAS* genes in 56 GGNs from 24 patients, and none of these patients showed identical *EGFR* or *KRAS* gene status. Thus, they suggested that these GGNs arise as independent events rather than intrapulmonary spread or systemic metastasis. Subsequently, Wu et al. conducted comprehensive and concurrent analysis of eight oncogenic driver genes (*EGFR, KRAS, HER2, BRAF, PIK3CA, ALK, ROS1,* and *RET*) in 72 lesions (60 GGNs) from 35 patients and found a high discordance rate of 68.6% (24 of 35) in the whole population and 80% (24 of 30) in patients harboring at least one of the detected driver mutations [16]. The results also showed that eight patients harbored the same mutations (5 in EGFR L858R and 3 in EGFR 19 deletion). Based on the high discrepancy of somatic driver mutations, they concluded that many of GGNs likely have developed as independent primaries rather than metastatic disease, and resection might be a proper option for multiple GGNs. A larger cohort including 159 GGNs from 78 patients was used to investigate the EGFR mutation status between different lesions, and a high discordance rate of 92.1% (35/38) in patients harboring EGFR mutation was observed [17]. Although three patients had identical EGFR mutation status, the authors concluded that multiple GGN lesions seem to arise from different origins and developed independently. Recently, Li et al. performed WES on 14 GGNs from two patients (8 and 6 lesions in one patient, respectively) to investigate whether multiple GGNs may represent intrapulmonary metastases [18]. Based on the shared mutations, especially those occurring in rarely reported genes, two of the multiple GGNs in each patient were found to be clonally related, indicative of intrapulmonary metastasis. This was the first and sole report demonstrating early intrapulmonary metastasis among GGNs. However, this conclusion was made based only on the shared mutations, which is not sufficient to prove the clonal relationships between tumors. Moreover, intrapulmonary metastatic tumors may have no shared mutations because of progressive accumulation of genetic alterations.

In a word, all of these studies were based on shared mutations, either using targeted sequencing or WES. Nevertheless, some other methods have been used to distinguish independent primary tumors from intrapulmonary metastasis, including CNV profiling [19–21], DNA rearrangements [22], and clone evolution analysis [23]. In this study, we combined the results of different methods to identify the clonal relationships between GGNs from each patient, including CNV profiling, shared somatic mutations, 6 substitution profiling, 96 substitution profiling and clone evolution diagram. As a result, 2 of 19 patients were identified as having possible intrapulmonary metastatic GGNs, suggesting that early metastasis may exist in GGNs. Clinical evidence of metastatic GGN from lung [8, 9] and other organs [10–14] has also been reported. According to the Fleischner guidelines [24], multiple GGNs are considered as multiple primaries, and are routinely treated as independent primary tumors. The findings that metastasis exists in multiple GGNs may aid stratification of patients with GGNs at the genetic level, and guide subsequent individualized treatment of patients with GGNs and follow-up.

Moreover, as the cause of GGNs is an issue that remains unresolved [3], we attempted to identify the factors causing GGN based on 96 substitutions. We found Signature 1, 3 and 6 had the highest proportions among the 30 COSMIC mutational signatures. Signature 1, characterized by *C* > *T* transition, correlates with age of cancer diagnosis, and has been found in all cancer types and in most cancer samples. Signature 3 is associated with failure of DNA double-strand break repair by homologous recombination. Signature 6 is associated with defective DNA mismatch repair and is found in microsatellite unstable tumors. In this study, 12 of 19 (63.2%) patients harbored mutations in DNA damage repair (DDR) genes, suggesting that GGN may be associated with deficient DDR pathway (Fig. S6). However, no recurrent mutated DDR genes were found. In addition, the prevalence of EGFR mutations and TP53 mutations were 21.7% (13/60) and 1.7% (1/60), respectively, indicating that EGFR mutation is an earlier event than TP53 mutation, although both of them are early events in lung cancer [25].

In conclusion, our findings suggest that intrapulmonary metastasis exist in multiple GGNs, but the number of GGNs was not associated with the probability of metastasis. Application of genomic profiling in the clinical setting may prove to be important for precise management of patients with multiple GGNs.

## Supplementary Information

Below is the link to the electronic supplementary material.

Supplementary file1 (DOCX 6977 KB)
