# Supplementary material for: Utility of whole exome sequencing analysis in differentiating intrapulmonary metastatic multiple ground-glass nodules (GGNs) from multiple primary GGNs
Source: Int J Clin Oncol. 2022 Feb 16;27(5):871–81. doi: 10.1007/s10147-022-02134-8 (PMC9023437; doi:10.1007/s10147-022-02134-8)
Supplement: Supplementary file 1 — Supplementary file1 (DOCX 6977 KB) [file 10147_2022_2134_MOESM1_ESM.docx]

Supplementary materials

**Supplementary Table 1. Demographic and Clinicopathological Characteristics and Follow-up Outcomes**

| Patient ID | Sex/  Age | Number of lesions | Tumor Size  (cm) | Surgery | Histologic type | Stage | DFS (months) | Postoperative metastasis | Follow-up duration (months) | Outcome |
| --- | --- | --- | --- | --- | --- | --- | --- | --- | --- | --- |
| P01 | F/27 | 2 | 0.46, 0.18 | Segmentectomy | MIA, AIS | Ia1 | 16 | no | 16 | alive |
| P02 | F/32 | 2 | 0.38, 0.15 | Wedge Resection | MIA, MIA | Ia1 | 16 | no | 16 | alive |
| P03 | F/33 | 2 | 0.31, 0.11 | Segmentectomy/Wedge Resection | AIS, AIS | - | 16 | no | 16 | alive |
| P04 | F/42 | 2 | 0.22, 0.10 | Lobectomy/Wedge Resection | MIA, MIA | Ia1 | 17 | no | 17 | alive |
| P05 | M/26 | 2 | 0.34, 0.12 | Segmentectomy/Wedge Resection | MIA, MIA | Ia1 | 16 | no | 16 | alive |
| P06 | F/48 | 2 | 0.38, 0.14 | Segmentectomy | LUAD, MIA | Ia1 | 15 | no | 15 | alive |
| P07 | M/58 | 2 | 1.05, 0.05 | Segmentectomy | MIA, AIS | Ia1 | 13 | no | 13 | alive |
| P08 | F/42 | 2 | 0.43, 0.18 | Wedge Resection | MIA, MIA | Ia1 | 15 | no | 15 | alive |
| P09 | F/47 | 2 | 0.38, 0.15 | Segmentectomy | MIA, AIS | Ia1 | 19 | no | 19 | alive |
| P10 | F/48 | 2 | 0.32, 0.12 | Lobectomy | MIA, MIA | Ia1 | 15 | no | 15 | alive |
| P11 | M/27 | 2 | 0.34, 0.10 | Wedge Resection | AIS, MIA | Ia1 | 15 | no | 15 | alive |
| P12 | M/49 | 3 | 0.45, 0.19, 0.07 | Segmentectomy/Wedge Resection | MIA, MIA, MIA | Ia1 | 16 | no | 16 | alive |
| P13 | F/49 | 3 | 0.75, 0.54, 0.18 | Lobectomy/Wedge Resection | MIA, MIA, MIA | Ia1 | 16 | no | 16 | alive |
| P14 | M/54 | 3 | 0.48, 0.32, 0.10 | Wedge Resection | AIS, AIS, AIS | - | 16 | no | 16 | alive |
| P15 | F/66 | 3 | 6.18, 2.64, 0.29 | Lobectomy/Wedge Resection | LUAD, MIA, MIA | Ia3 | 19 | no | 19 | alive |
| P16 | F/55 | 4 | 0.99, 0.18, 0.15, 0.07 | Segmentectomy/Wedge Resection | MIA, MIA, MIA, MIA | Ia1 | 16 | no | 16 | alive |
| P17 | F/53 | 5 | 0.43, 0.25, 0.13, 0.08, 0.05 | Lobectomy/Wedge Resection | MIA, AIS, MIA, AIS, MIA | Ia1 | 17 | no | 17 | alive |
| P18 | F/57 | 5 | 1.24, 0.32, 0.14, 0.10, 0.05 | Wedge Resection | MIA, MIA, MIA, MIA, MIA | Ia1 | 17 | no | 17 | alive |
| P19 | F/50 | 12 | 0.97, 0.55, 0.38, 0.32, 0.20, 0.11, 0.10, 0.09, 0.09, 0.07, 0.06, 0.04 | Wedge Resection | All MIA | Ia1 | 16 | no | 16 | alive |

MIA, minimally invasive adenocarcinoma; AIS, adenocarcinoma in situ; LUAD, [lung adenocarcinoma](javascript:;).

“-” means unknown.

**Supplementary Table 2.** The correlation coefficients between ground-glass nodules (GGNs) calculated by 96 substitution profiles.

|  |  | T1 | T2 | T3 | T4 | T5 | T6 | T7 | T8 | T9 | T10 | T11 | T12 |
| --- | --- | --- | --- | --- | --- | --- | --- | --- | --- | --- | --- | --- | --- |
| P01 | T2 | -0.07 |  |  |  |  |  |  |  |  |  |  |  |
| P02 | T2 | 0.61 |  |  |  |  |  |  |  |  |  |  |  |
| P03 | T2 | 0.17 |  |  |  |  |  |  |  |  |  |  |  |
| P04 | T2 | -0.09 |  |  |  |  |  |  |  |  |  |  |  |
| P05 | T2 | 0.33 |  |  |  |  |  |  |  |  |  |  |  |
| P06 | T2 | 0.89 |  |  |  |  |  |  |  |  |  |  |  |
| P07 | T2 | 0.36 |  |  |  |  |  |  |  |  |  |  |  |
| P08 | T2 | 0.72 |  |  |  |  |  |  |  |  |  |  |  |
| P09 | T2 | 0.09 |  |  |  |  |  |  |  |  |  |  |  |
| P10 | T2 | 0.56 |  |  |  |  |  |  |  |  |  |  |  |
| P11 | T2 | 0.71 |  |  |  |  |  |  |  |  |  |  |  |
| P12 | T2 | 0.14 |  |  |  |  |  |  |  |  |  |  |  |
|  | T3 | 0.09 |  |  |  |  |  |  |  |  |  |  |  |
| P13 | T2 | 0.71 |  |  |  |  |  |  |  |  |  |  |  |
|  | T3 | 0.06 | 0.01 |  |  |  |  |  |  |  |  |  |  |
| P14 | T2 | 0.28 |  |  |  |  |  |  |  |  |  |  |  |
|  | T3 | 0.32 | 0.26 |  |  |  |  |  |  |  |  |  |  |
| P15 | T2 | 0.95 |  |  |  |  |  |  |  |  |  |  |  |
|  | T3 | 0.97 | 0.93 |  |  |  |  |  |  |  |  |  |  |
| P16 | T2 | -0.06 |  |  |  |  |  |  |  |  |  |  |  |
|  | T3 | 0.84 | -0.04 |  |  |  |  |  |  |  |  |  |  |
|  | T4 | 0.50 | 0.10 | 0.51 |  |  |  |  |  |  |  |  |  |
| P17 | T2 | 0.10 |  |  |  |  |  |  |  |  |  |  |  |
|  | T3 | 0.31 | 0.21 |  |  |  |  |  |  |  |  |  |  |
|  | T4 | 0.46 | 0.11 | 0.23 |  |  |  |  |  |  |  |  |  |
|  | T5 | 0.56 | 0.16 | 0.26 | 0.65 |  |  |  |  |  |  |  |  |
| P18 | T2 | -0.07 |  |  |  |  |  |  |  |  |  |  |  |
|  | T3 | 0.23 | 0.00 |  |  |  |  |  |  |  |  |  |  |
|  | T4 | 0.29 | 0.18 | 0.24 |  |  |  |  |  |  |  |  |  |
|  | T5 | 0.20 | 0.64 | 0.14 | 0.18 |  |  |  |  |  |  |  |  |
| P19 | T2 | 0.68 |  |  |  |  |  |  |  |  |  |  |  |
|  | T3 | 0.48 | 0.38 |  |  |  |  |  |  |  |  |  |  |
|  | T4 | 0.56 | 0.67 | 0.54 |  |  |  |  |  |  |  |  |  |
|  | T5 | 0.72 | 0.64 | 0.49 | 0.62 |  |  |  |  |  |  |  |  |
|  | T6 | 0.63 | 0.60 | 0.26 | 0.56 | 0.56 |  |  |  |  |  |  |  |
|  | T7 | 0.33 | 0.40 | 0.07 | 0.22 | 0.31 | 0.27 |  |  |  |  |  |  |
|  | T8 | 0.43 | 0.34 | 0.30 | 0.34 | 0.31 | 0.40 | 0.12 |  |  |  |  |  |
|  | T9 | 0.57 | 0.43 | 0.18 | 0.33 | 0.38 | 0.58 | 0.12 | 0.40 |  |  |  |  |
|  | T10 | 0.76 | 0.63 | 0.47 | 0.56 | 0.78 | 0.67 | 0.43 | 0.38 | 0.53 |  |  |  |
|  | T11 | 0.84 | 0.81 | 0.49 | 0.69 | 0.77 | 0.64 | 0.40 | 0.47 | 0.47 | 0.75 |  |  |
|  | T12 | 0.30 | 0.31 | 0.19 | 0.27 | 0.27 | 0.30 | 0.19 | 0.36 | 0.23 | 0.25 | 0.35 |  |

Red represents Pearson coefficient >0.8, indicating a complete positive correlation.

**Supplementary Table 3.** The consistency of genetic characteristics for each tumor pair in patient 19.

| Patient ID | Tumor Pairs | CNV profiling | Shared somatic mutations | Six substitution profile | Ninety-six substitution profile | Clone evolution diagram |
| --- | --- | --- | --- | --- | --- | --- |
| P19 | T1 vs T2 | √ |  |  |  |  |
| P19 | T1 vs T3 | √ |  |  |  |  |
| P19 | T1 vs T4 | √ |  |  |  |  |
| P19 | T1 vs T5 | √ |  | √ |  |  |
| P19 | T1 vs T6 | √ |  |  |  |  |
| P19 | T1 vs T7 | √ |  |  |  |  |
| P19 | T1 vs T8 | √ |  |  |  |  |
| P19 | T1 vs T9 | √ |  |  |  |  |
| P19 | T1 vs T10 | √ | √ |  |  |  |
| P19 | T1 vs T11 | √ |  |  | √ |  |
| P19 | T1 vs T12 | √ |  |  |  |  |
| P19 | T2 vs T3 | √ |  |  |  |  |
| P19 | T2 vs T4 | √ |  |  |  |  |
| P19 | T2 vs T5 | √ |  |  |  |  |
| P19 | T2 vs T6 | √ |  |  |  |  |
| P19 | T2 vs T7 | √ | √ |  |  |  |
| P19 | T2 vs T8 | √ |  |  |  |  |
| P19 | T2 vs T9 | √ |  |  |  |  |
| P19 | T2 vs T10 | √ | √ |  |  |  |
| P19 | T2 vs T11 | √ |  |  | √ |  |
| P19 | T2 vs T12 | √ |  |  |  |  |
| P19 | T3 vs T4 | √ |  |  |  |  |
| P19 | T3 vs T5 | √ |  |  |  |  |
| P19 | T3 vs T6 | √ |  |  |  |  |
| P19 | T3 vs T7 | √ |  |  |  |  |
| P19 | T3 vs T8 | √ |  |  |  |  |
| P19 | T3 vs T9 | √ |  |  |  |  |
| P19 | T3 vs T10 | √ |  |  |  |  |
| P19 | T3 vs T11 | √ |  |  |  |  |
| P19 | T3 vs T12 | √ |  |  |  |  |
| P19 | T4 vs T5 | √ |  |  |  |  |
| P19 | T4 vs T6 | √ |  |  |  |  |
| P19 | T4 vs T7 | √ |  |  |  |  |
| P19 | T4 vs T8 | √ |  |  |  |  |
| P19 | T4 vs T9 | √ |  |  |  |  |
| P19 | T4 vs T10 | √ |  |  |  |  |
| P19 | T4 vs T11 | √ |  |  |  |  |
| P19 | T4 vs T12 | √ |  |  |  |  |
| P19 | T5 vs T6 | √ |  | √ |  |  |
| P19 | T5 vs T7 | √ |  |  |  |  |
| P19 | T5 vs T8 | √ |  |  |  |  |
| P19 | T5 vs T9 | √ |  |  |  |  |
| P19 | T5 vs T10 | √ |  |  |  |  |
| P19 | T5 vs T11 | √ |  |  |  |  |
| P19 | T5 vs T12 | √ |  |  |  |  |
| P19 | T6 vs T7 | √ |  |  |  |  |
| P19 | T6 vs T8 | √ |  |  |  |  |
| P19 | T6 vs T9 | √ |  |  |  |  |
| P19 | T6 vs T10 | √ |  |  |  |  |
| P19 | T6 vs T11 | √ |  |  |  |  |
| P19 | T6 vs T12 | √ |  |  |  |  |
| P19 | T7 vs T8 | √ |  |  |  |  |
| P19 | T7 vs T9 | √ |  |  |  |  |
| P19 | T7 vs T10 | √ | √ |  |  |  |
| P19 | T7 vs T11 | √ |  |  |  |  |
| P19 | T7 vs T12 | √ |  |  |  |  |
| P19 | T8 vs T9 | √ |  |  |  |  |
| P19 | T8 vs T10 | √ |  |  |  |  |
| P19 | T8 vs T11 | √ |  |  |  |  |
| P19 | T8 vs T12 | √ |  |  |  |  |
| P19 | T9 vs T10 | √ |  |  |  |  |
| P19 | T9 vs T11 | √ |  |  |  |  |
| P19 | T9 vs T12 | √ |  |  |  |  |
| P19 | T10 vs T11 | √ |  |  |  |  |
| P19 | T10 vs T12 | √ |  |  |  |  |
| P19 | T11 vs T12 | √ |  |  |  |  |

“√” indicates that the genetic characteristics of pairwise samples are consistent or similar.

**A**

**P03**


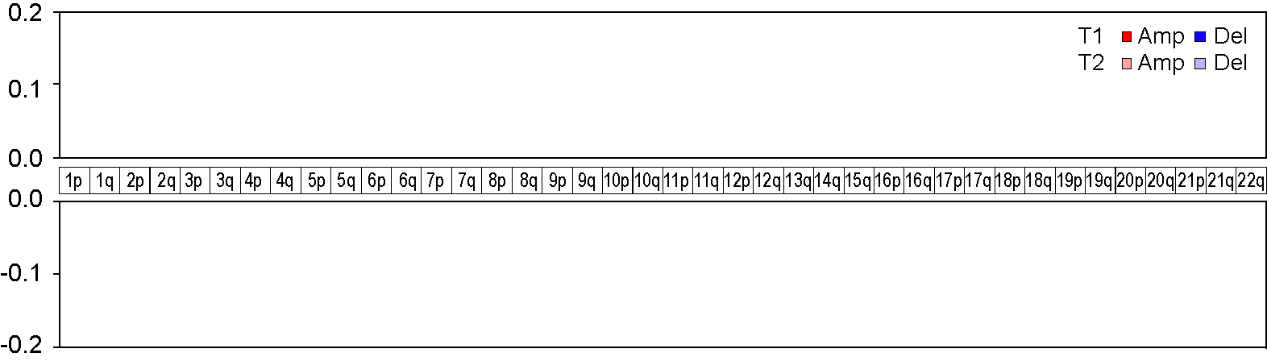


**B**

**P01**


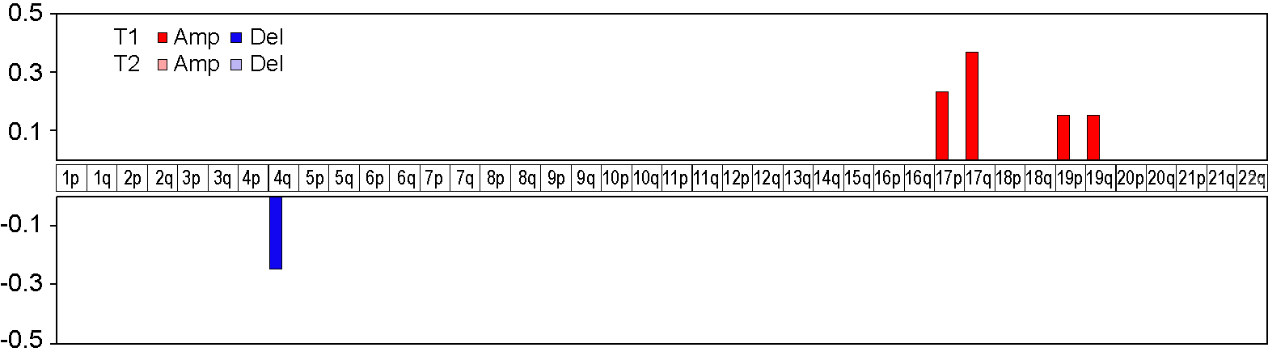


**P02**


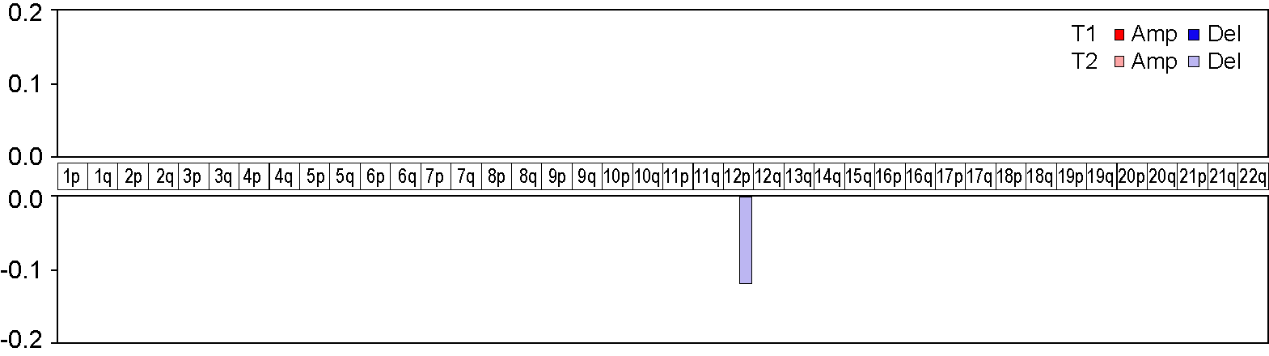


**P04**


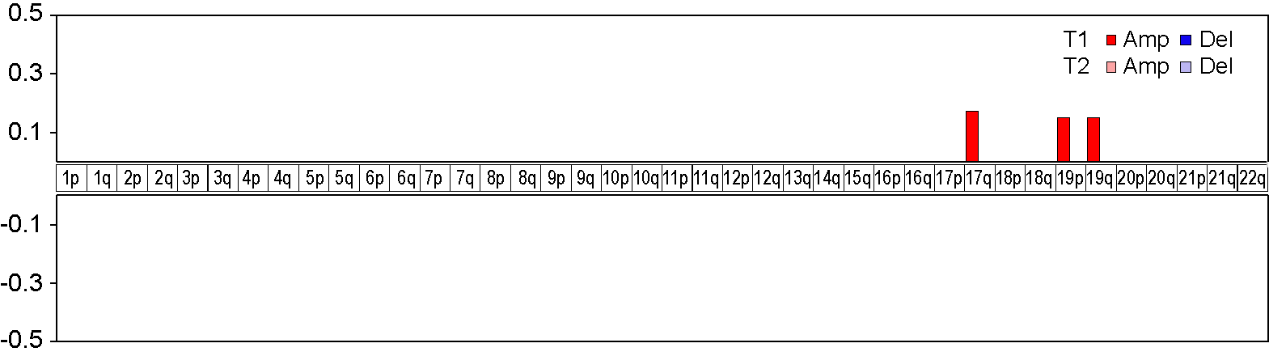


**P05**


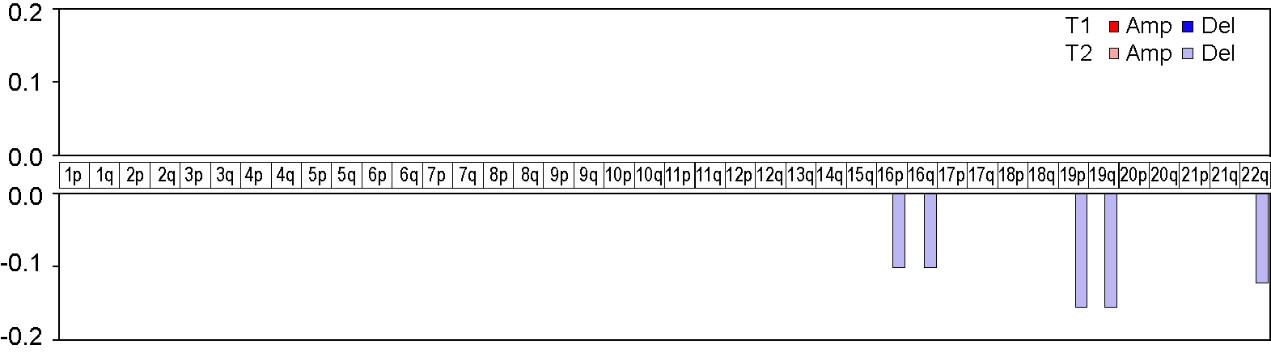


**P06**


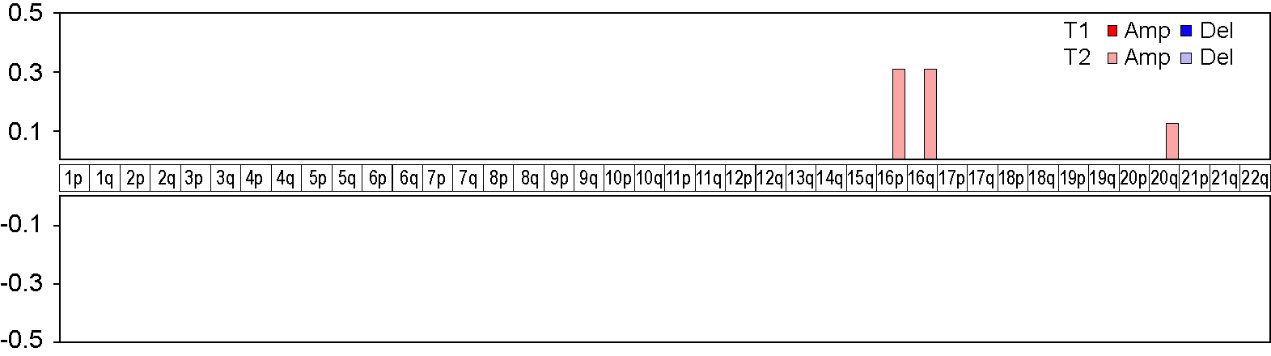


**P10**


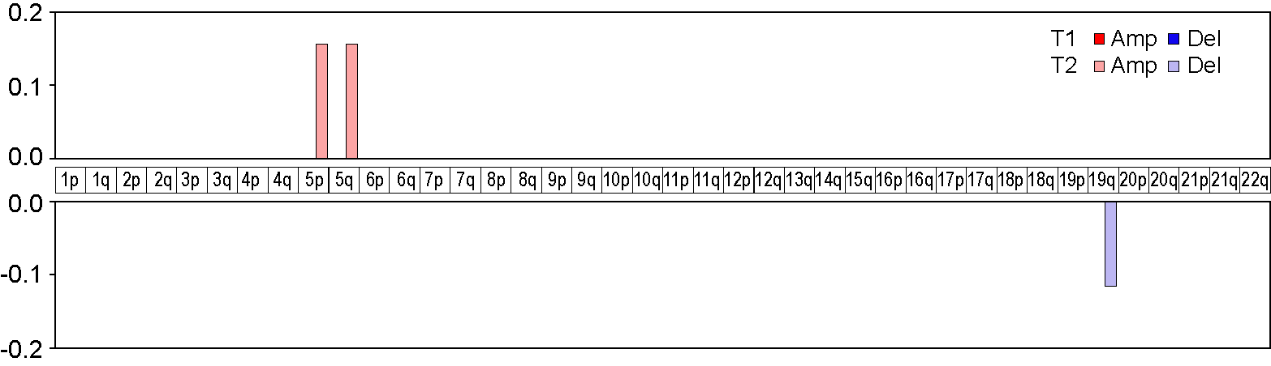


**P11**


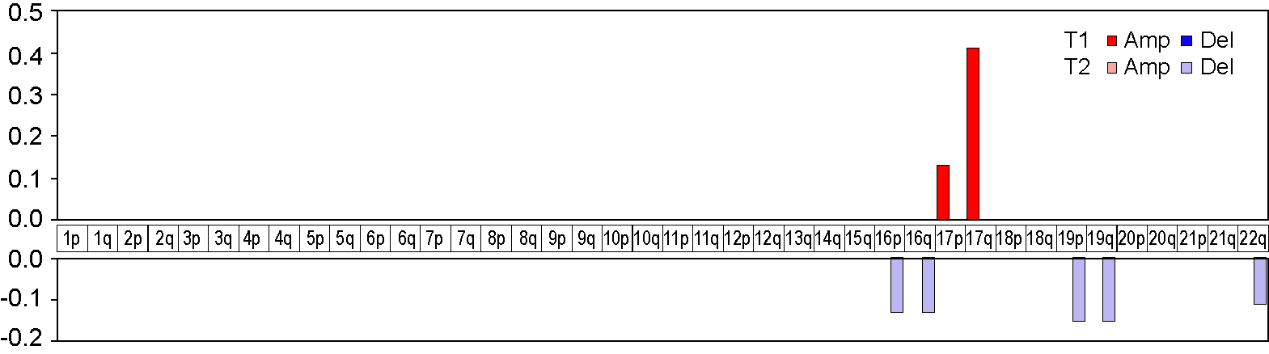


**P13**


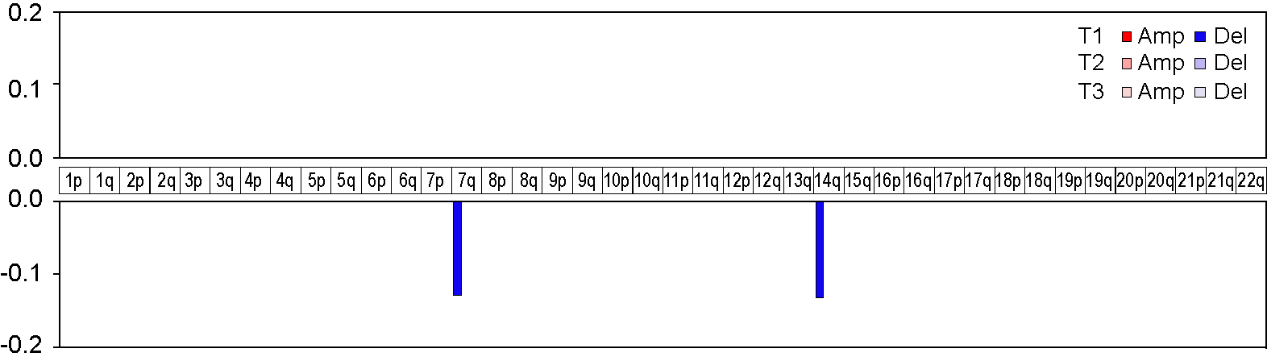


**C**

**P07
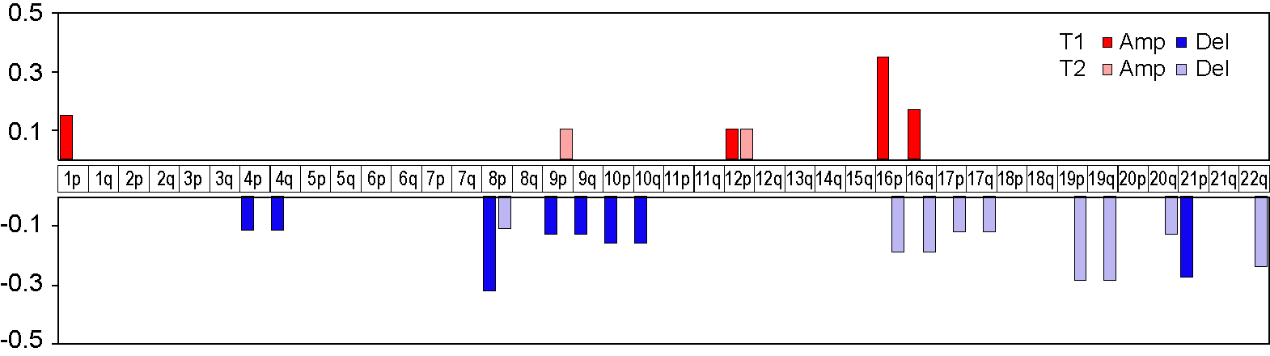
**

**P08
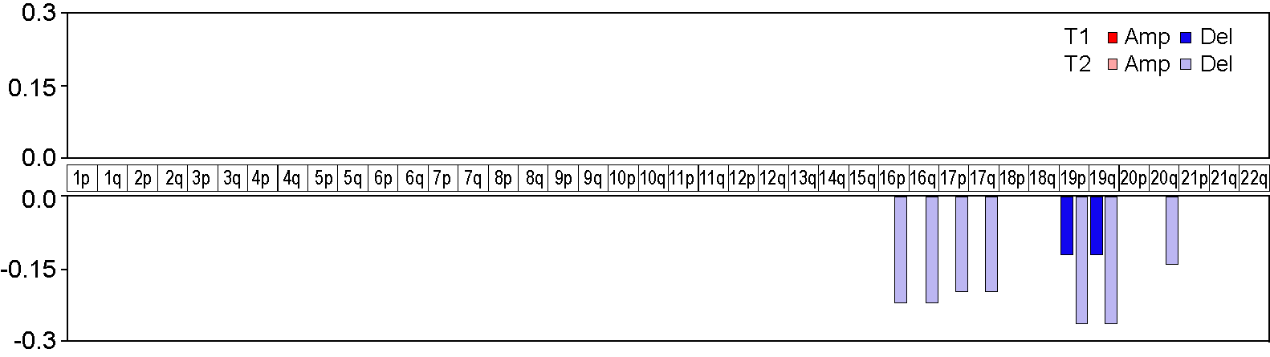
**

**P09
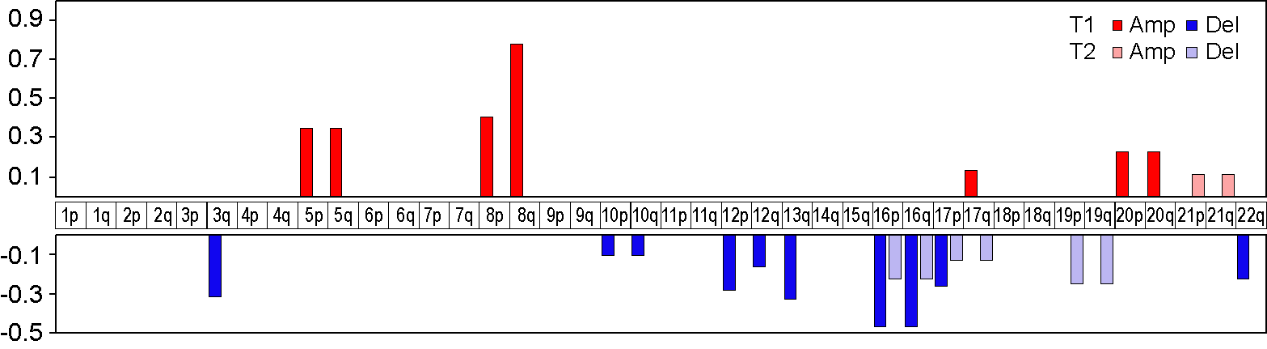
**

**P12
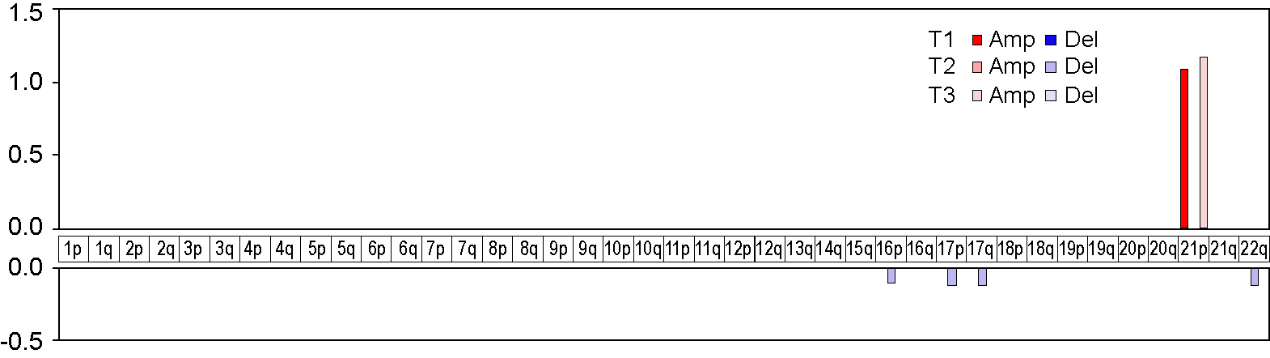
**

**P14
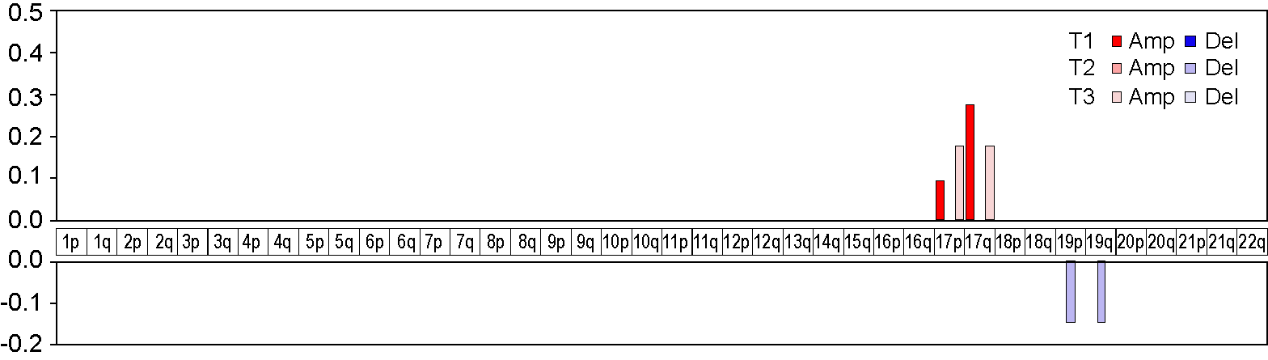
**

**P15
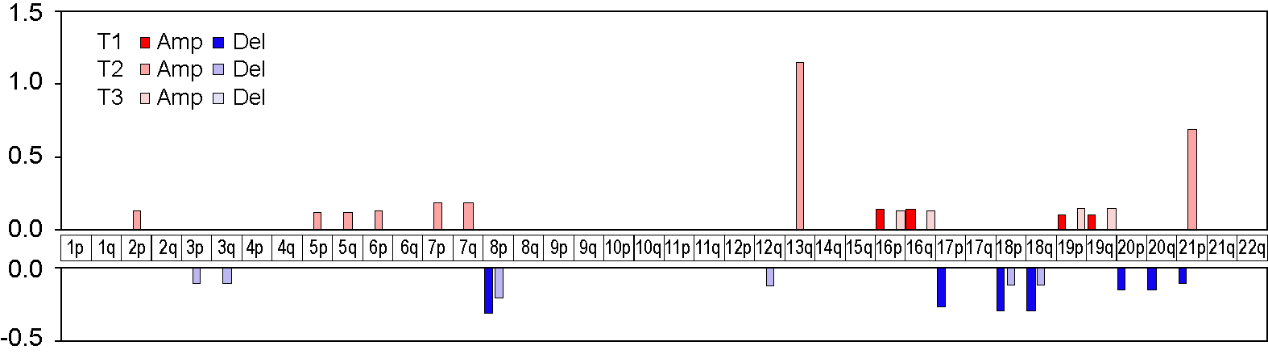
**

**P16
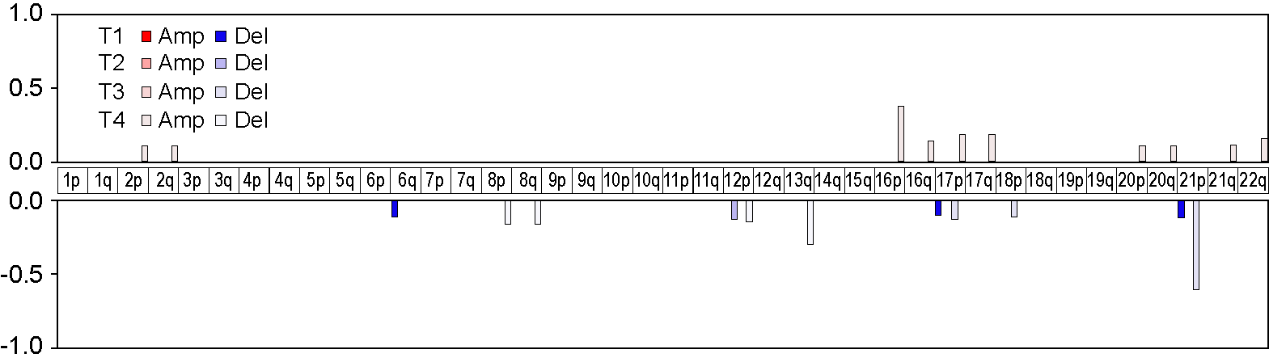
**

**P17
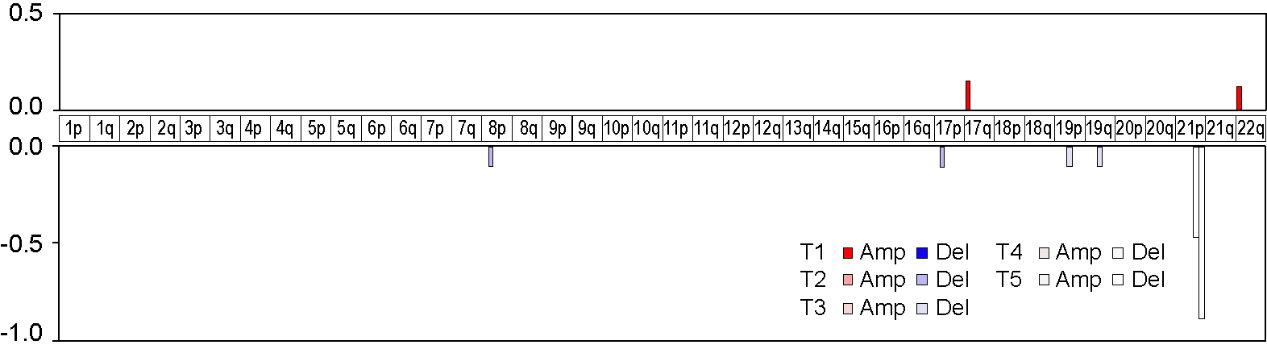
**

**P18
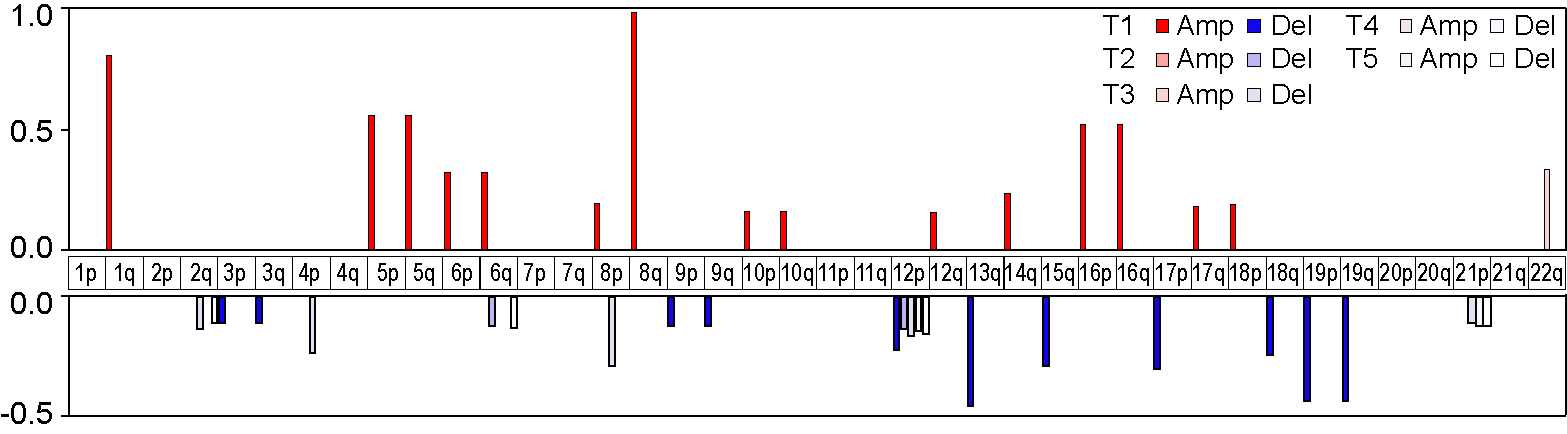
**

**P19
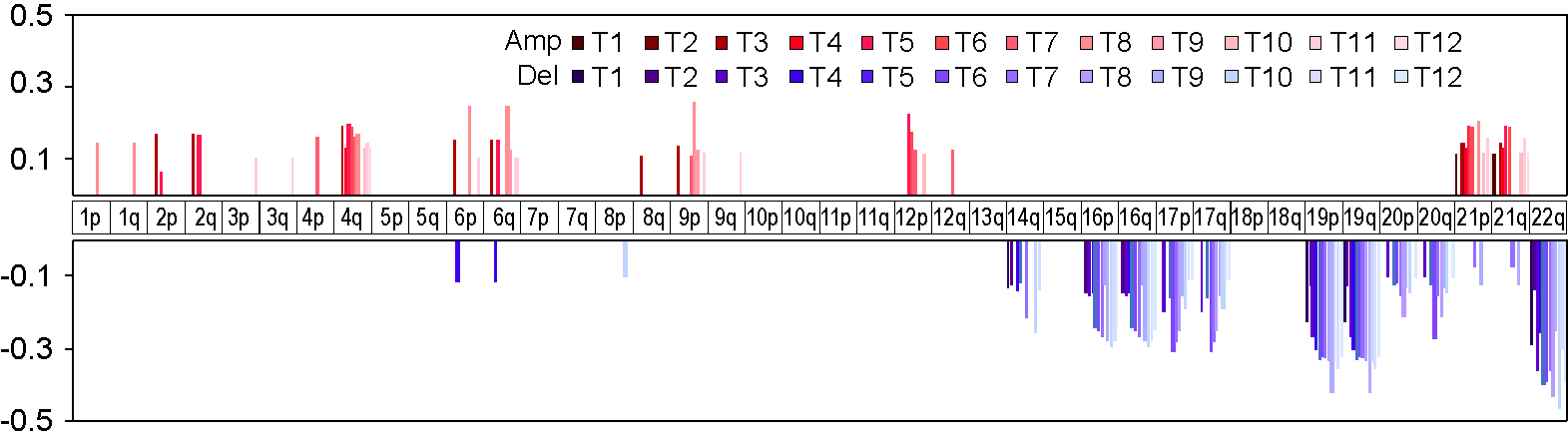
**

**Supplementary Figure 1.** Chromosome arm-level copy number variation (CNV) frequencies in each ground-glass nodule (GGN). (A)One patient with the absence of arm-level CNVs (P03). (B) Eight patients with different arm-level CNV profiles between GGNs. (C) Ten patients shared at least one arm-level CNV between GGNs from an individual patient. The top panel represents CNV amplifications (Amp) and the bottom panel represents CNV deletions (Del).


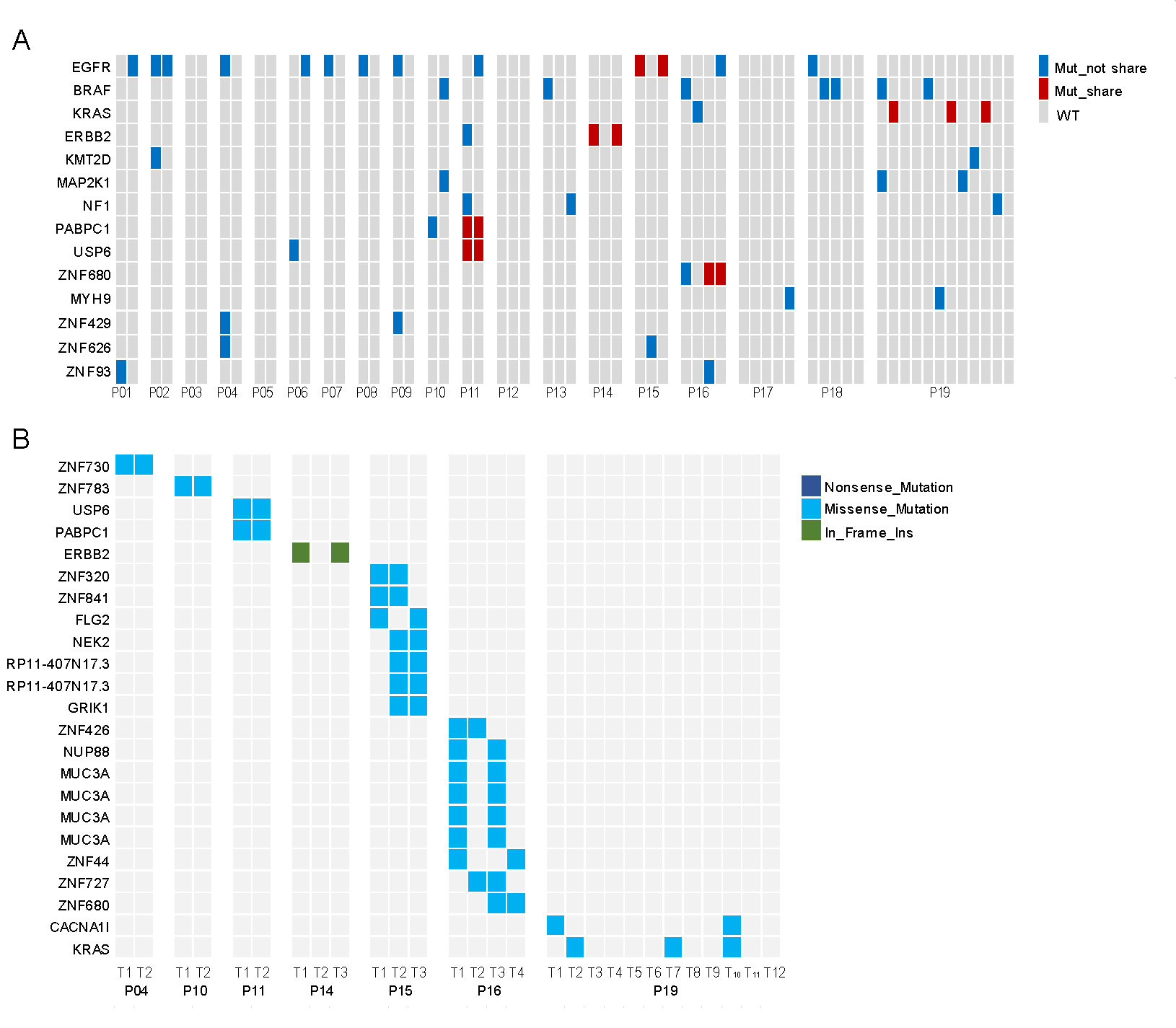


**Supplementary Figure 2.** Mutation profiles of ground-glass nodules (GGNs) in this cohort. (A) Mutated cancer-related genes in at least two samples. Mut_not share (blue) represents mutation that is not shared by GGNs in a patient. Mut_share (red) represents mutation that is shared by GGNs in a patient. WT (grey), wild type. (B) Genes with shared nonsynonymous mutations by GGNs in a patient.


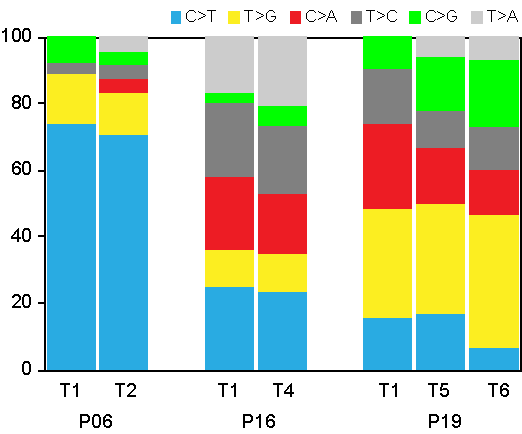


**Supplementary Figure 3.** Ground-glass nodules (GGNs) in three patients (P06, P16, P19) had similar six substitution profiles.

**P01**

**
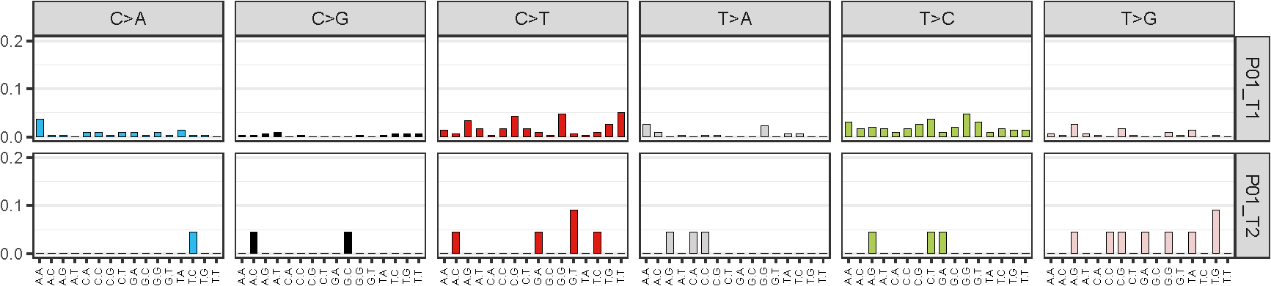
**

**P02
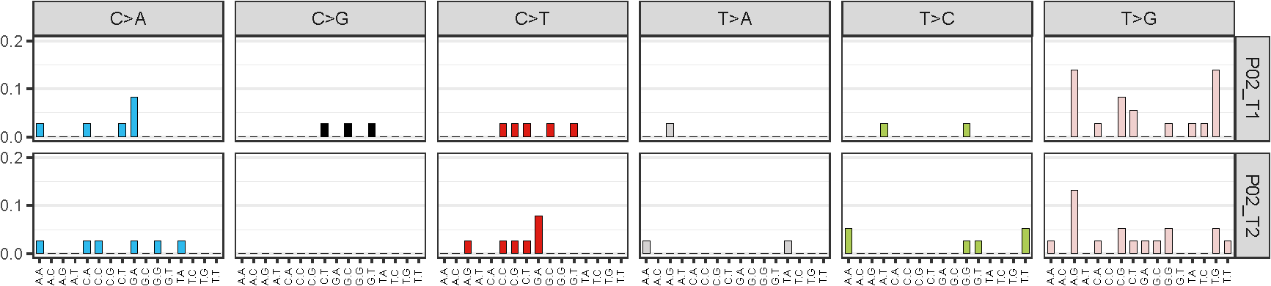
**

**P03
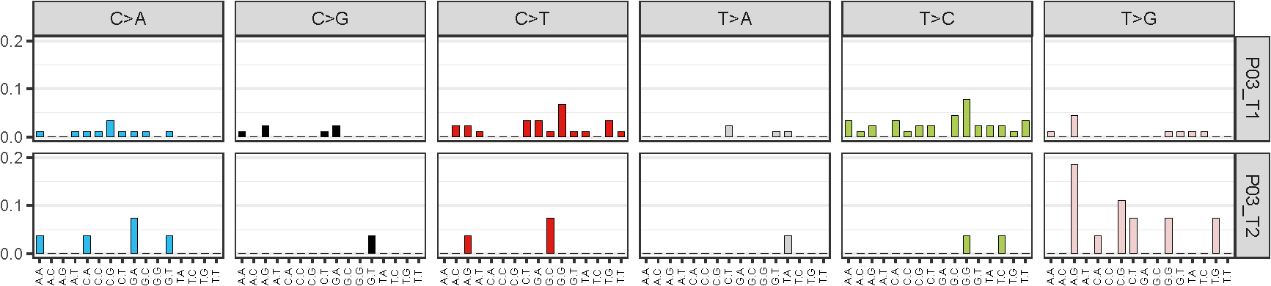
**

**P04
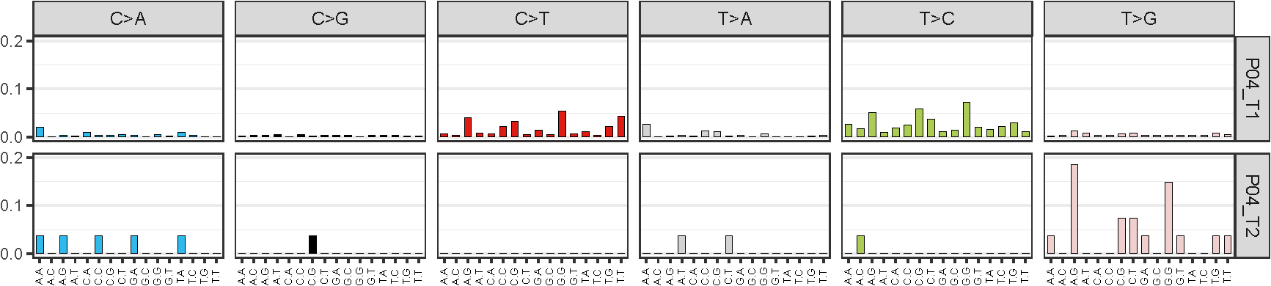
**

**P05
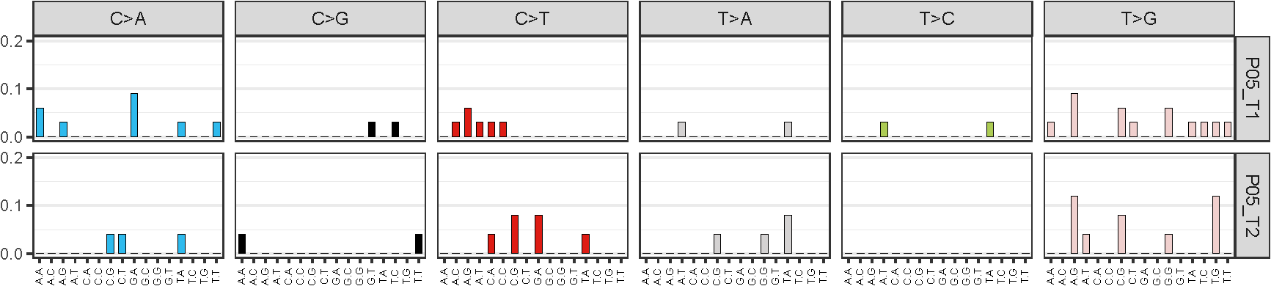
**

**P06
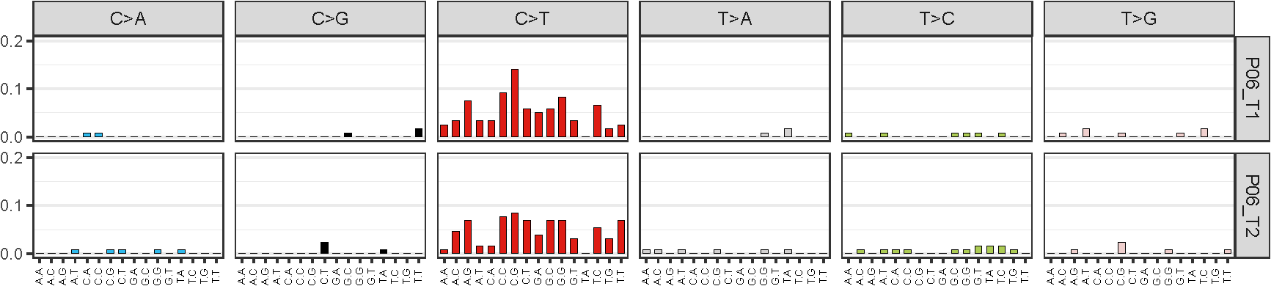
**

**P07
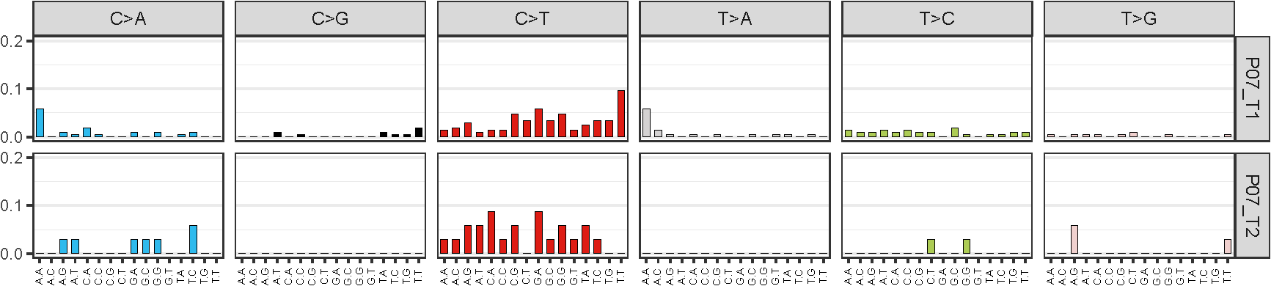
**

**P08
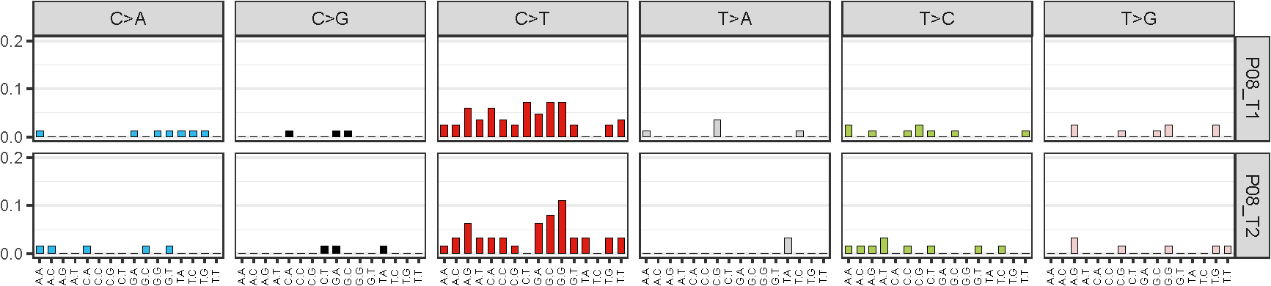
**

**P09
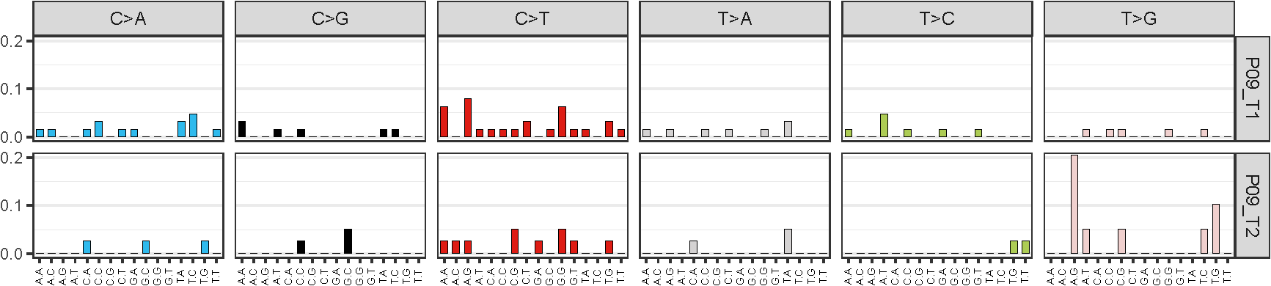
**

**P10
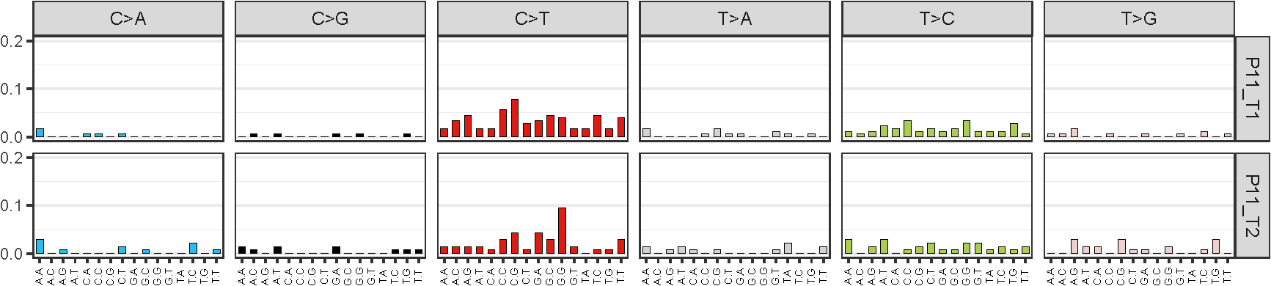
**

**P11
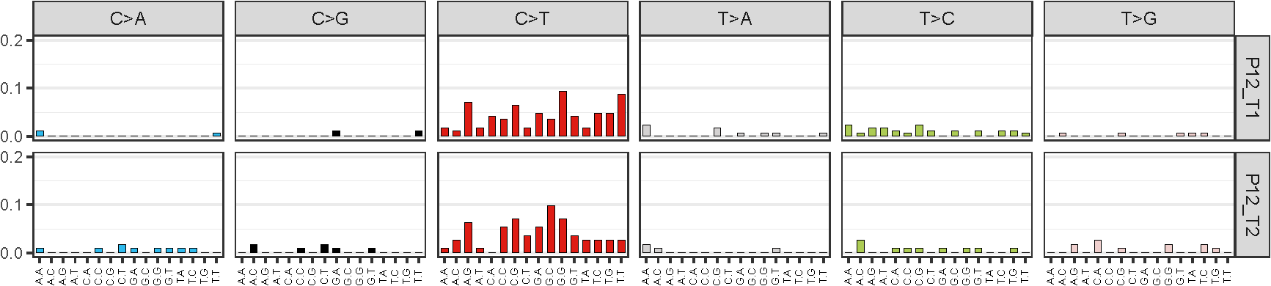
**

**P12
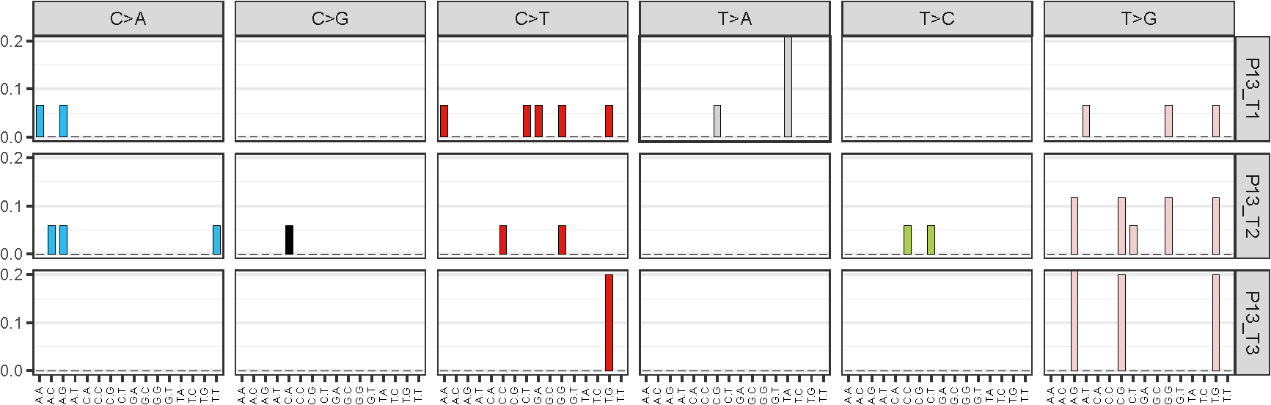
**

**P13
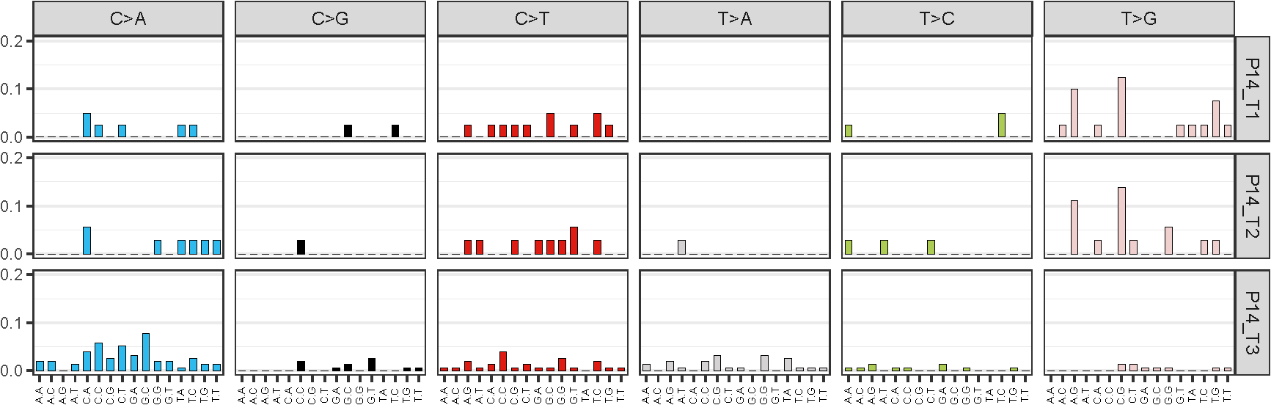
**

**P14
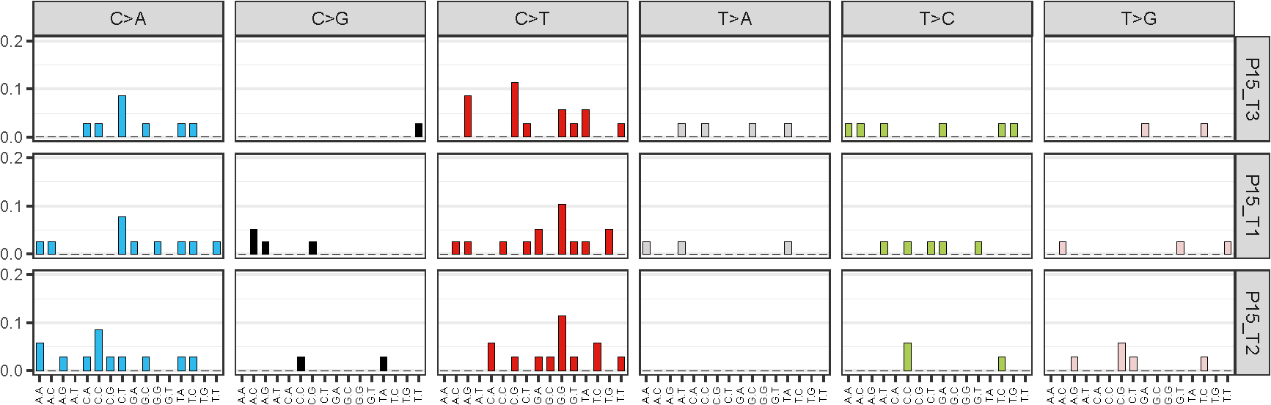
**

**P15
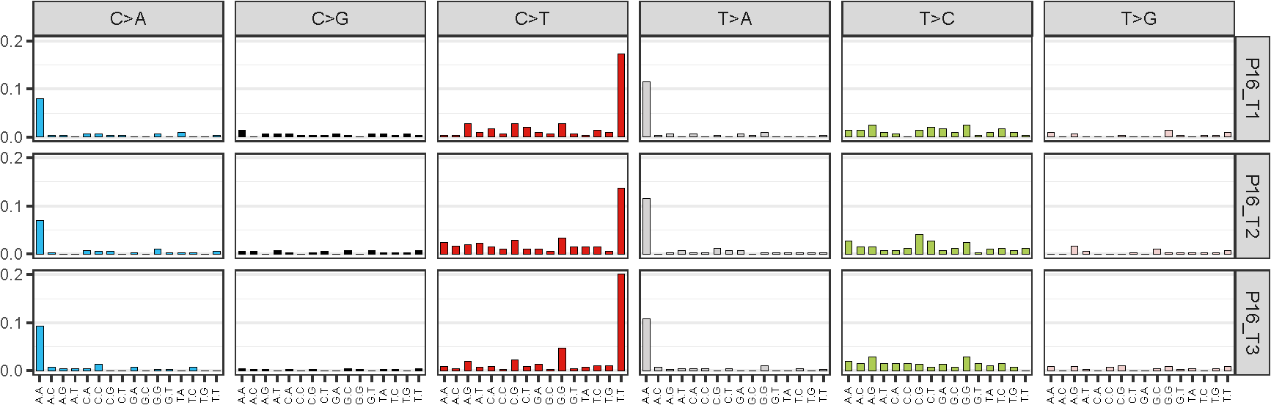
**

**P16
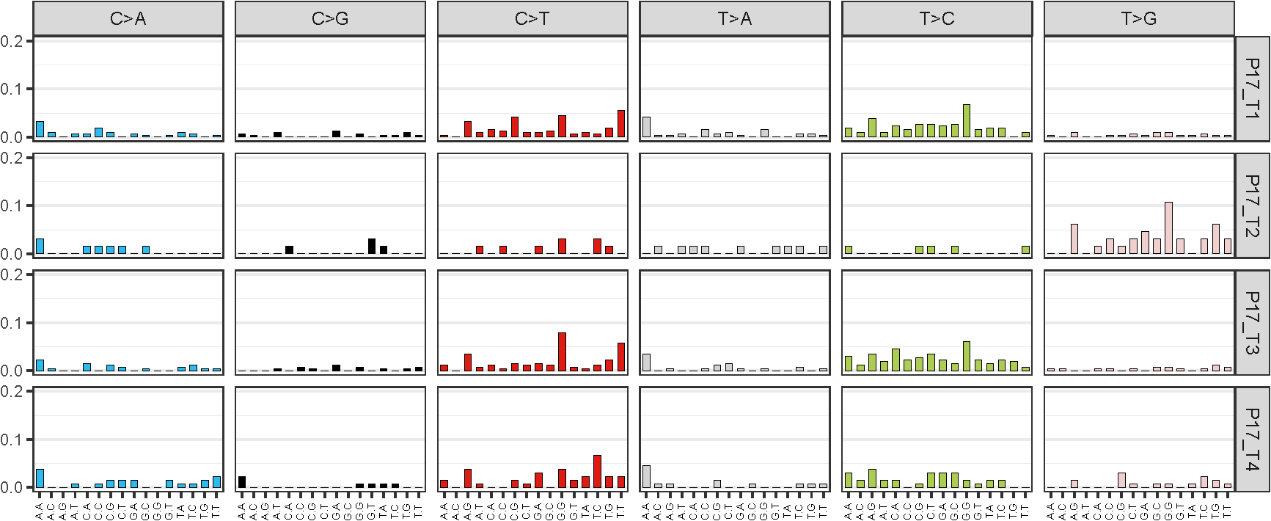
**

**P17
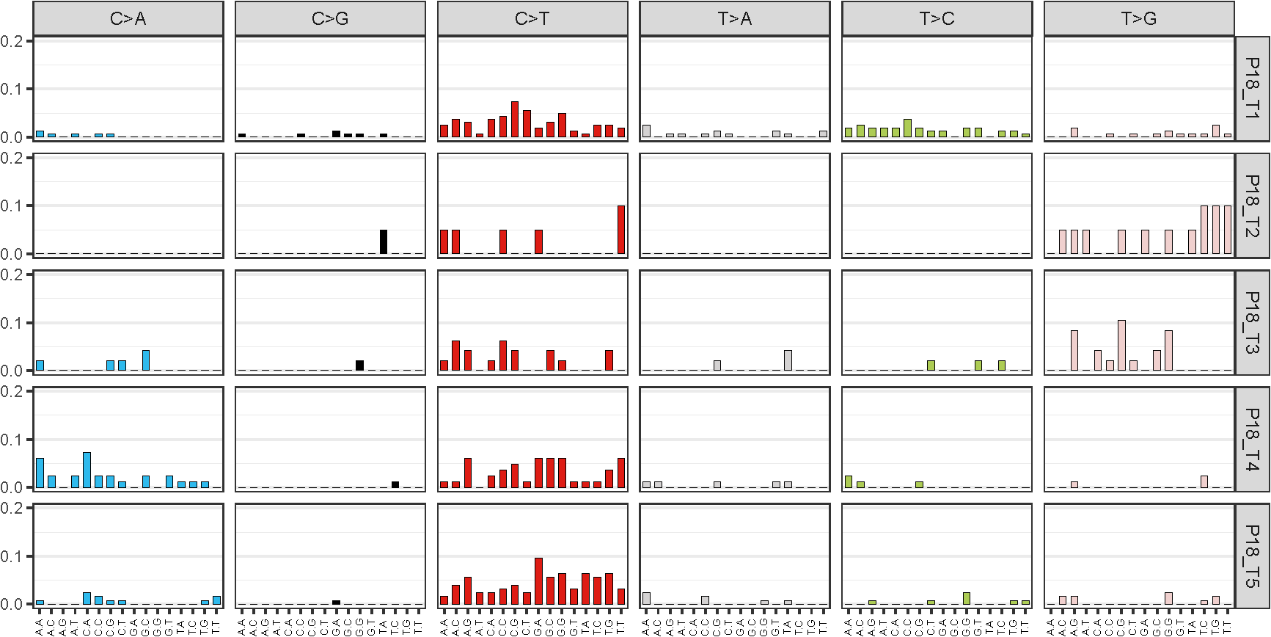
**

**P18
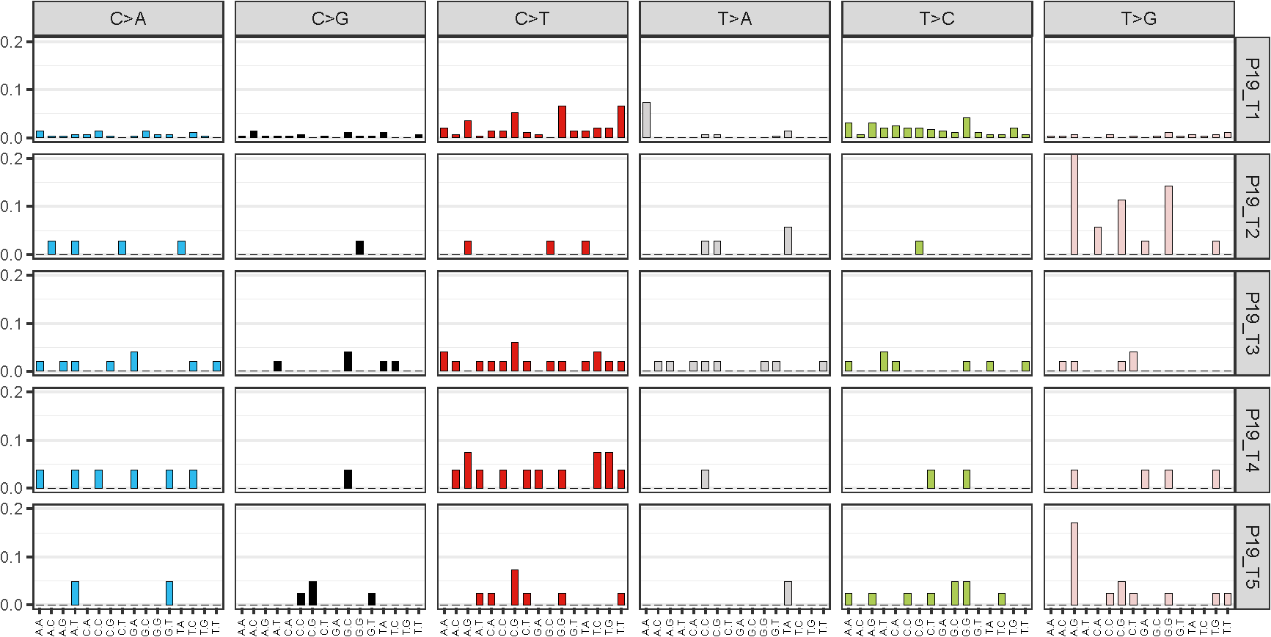
**

**P19
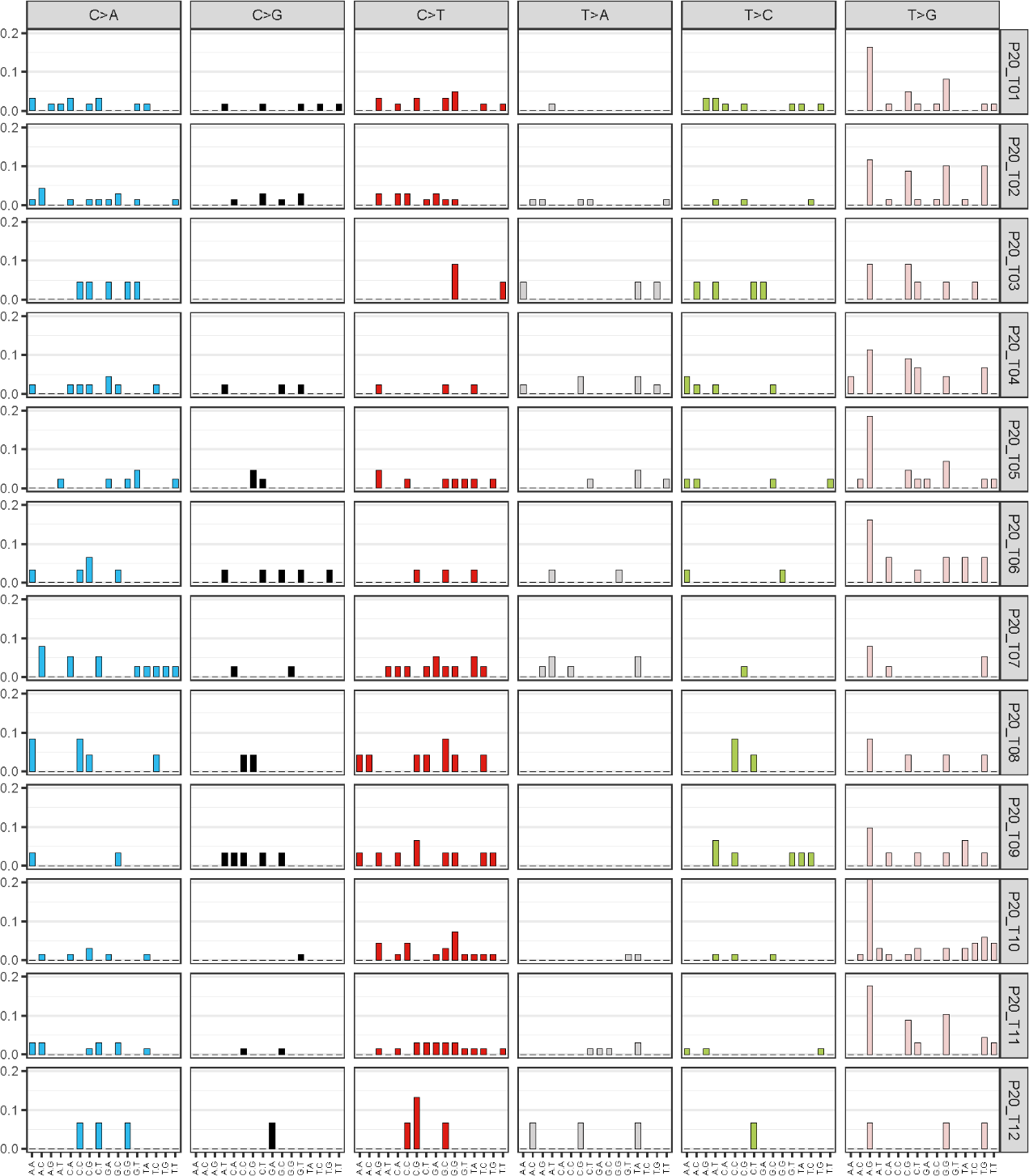
**

**Supplementary Figure 4.** The profiles of 96 substitutions for each ground-glass nodule (GGN).

**A**

**P01 P02 P03**

**
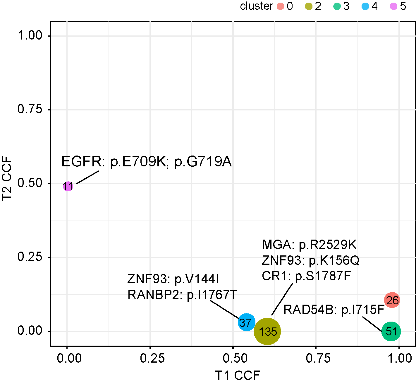

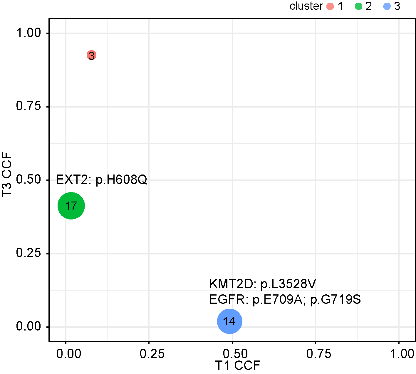

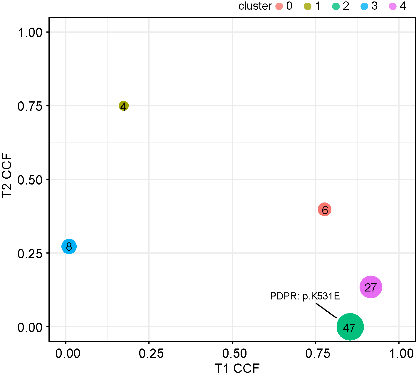
**

**P05 P07 P08**

**
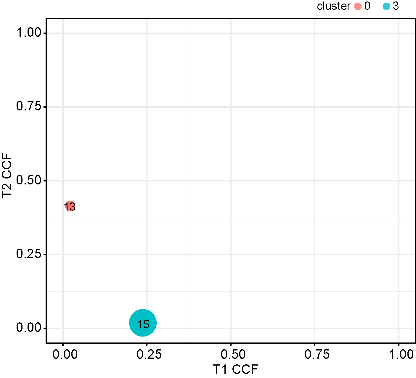

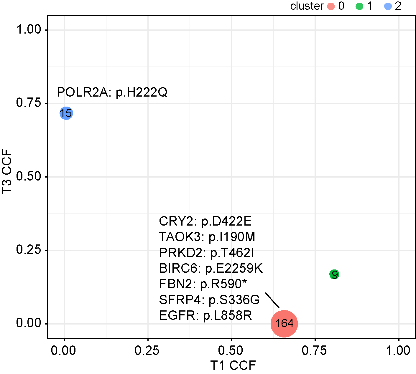

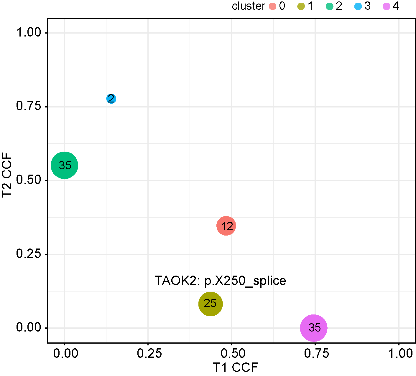
**

**P09 P10**

**
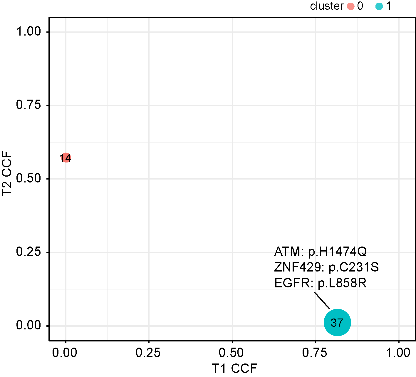

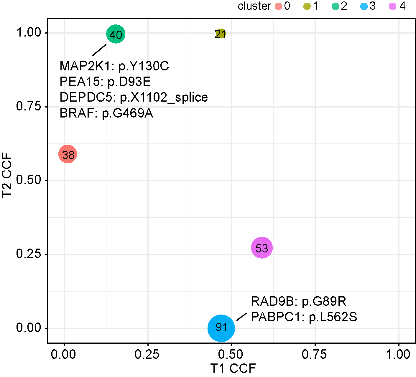
**

**P12**

**
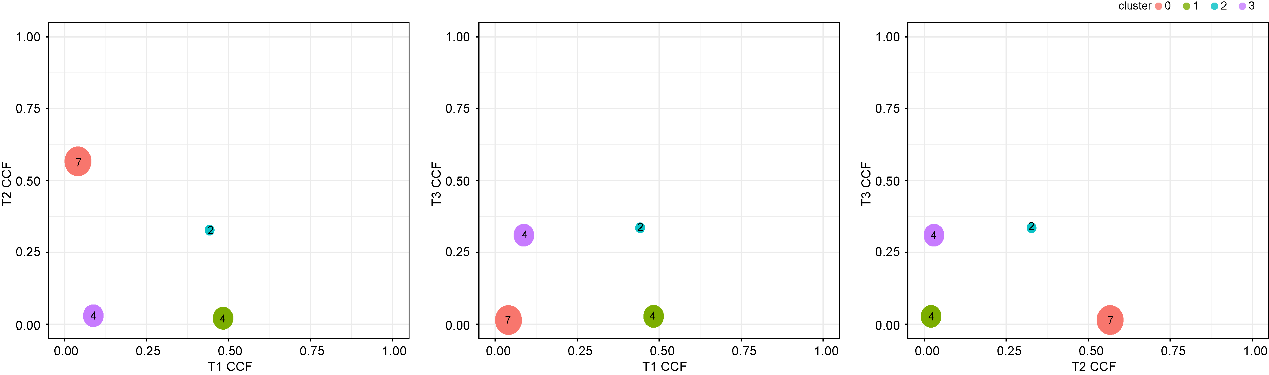
**

**P13**

**
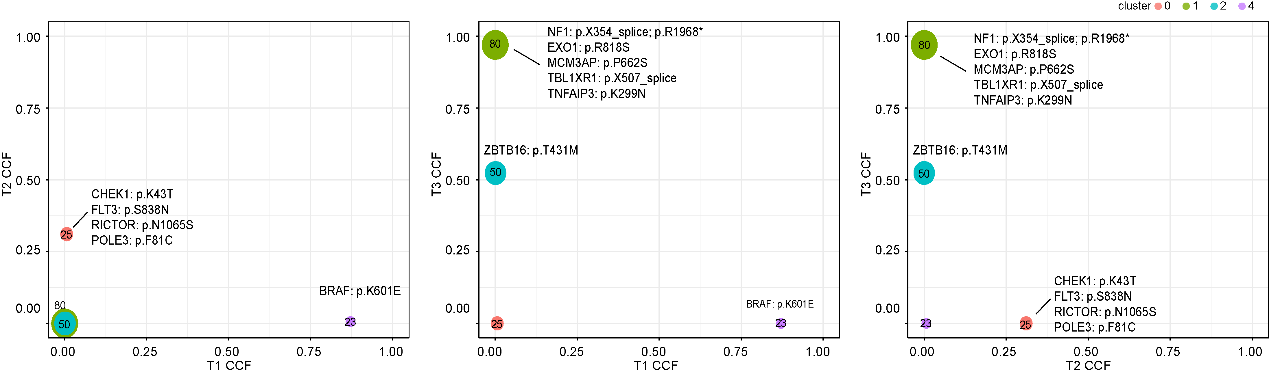
**

**P14**

**
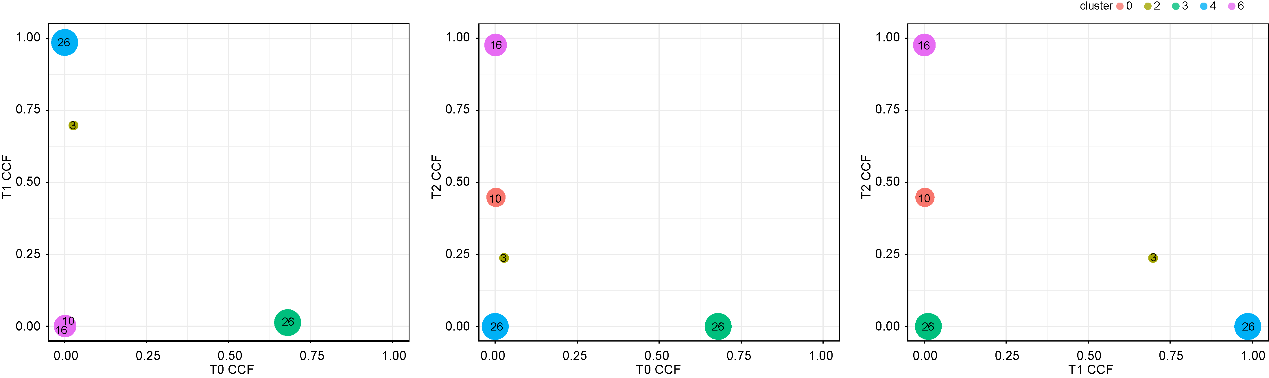
**

**P17**

**
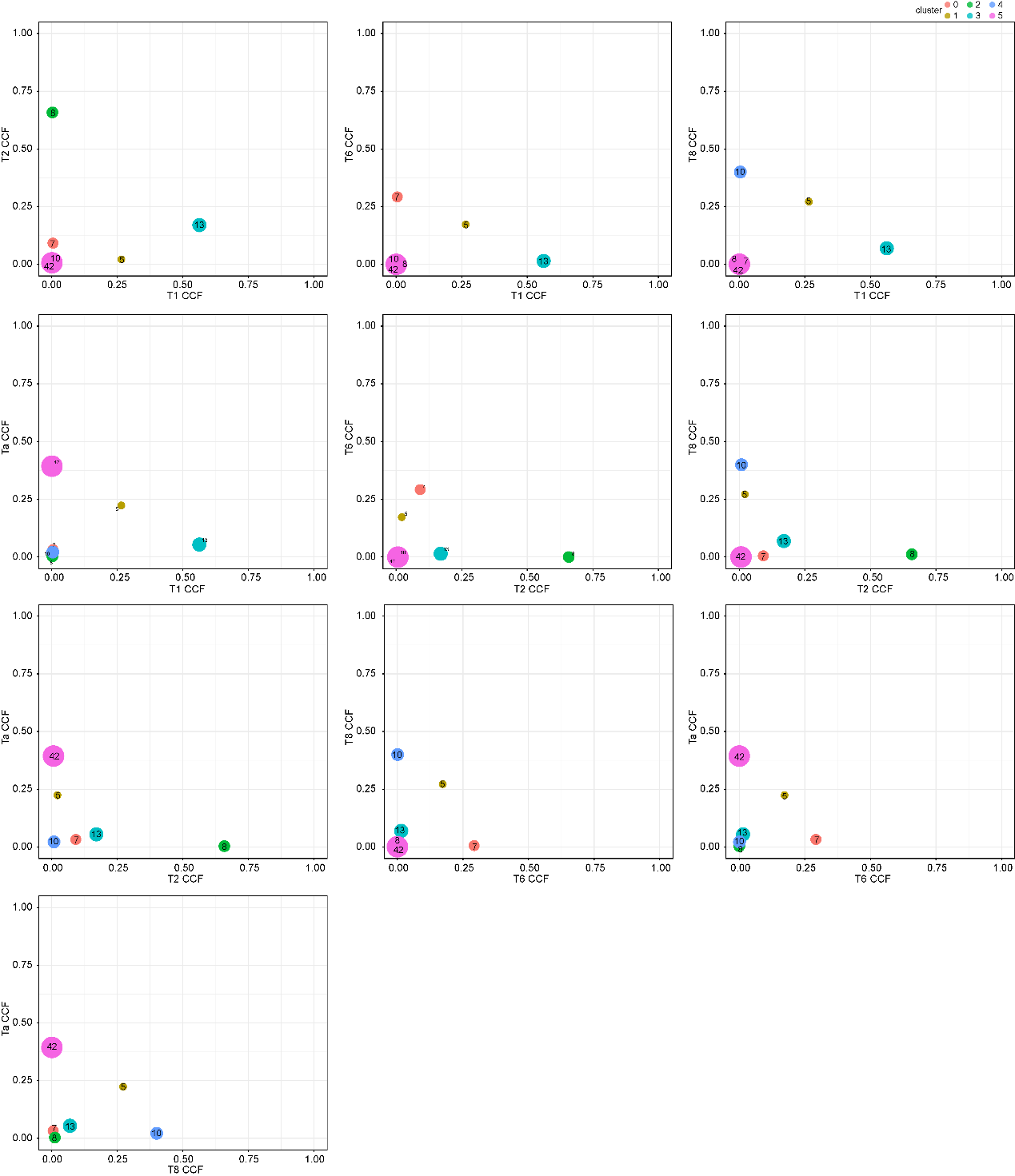
**

**P18
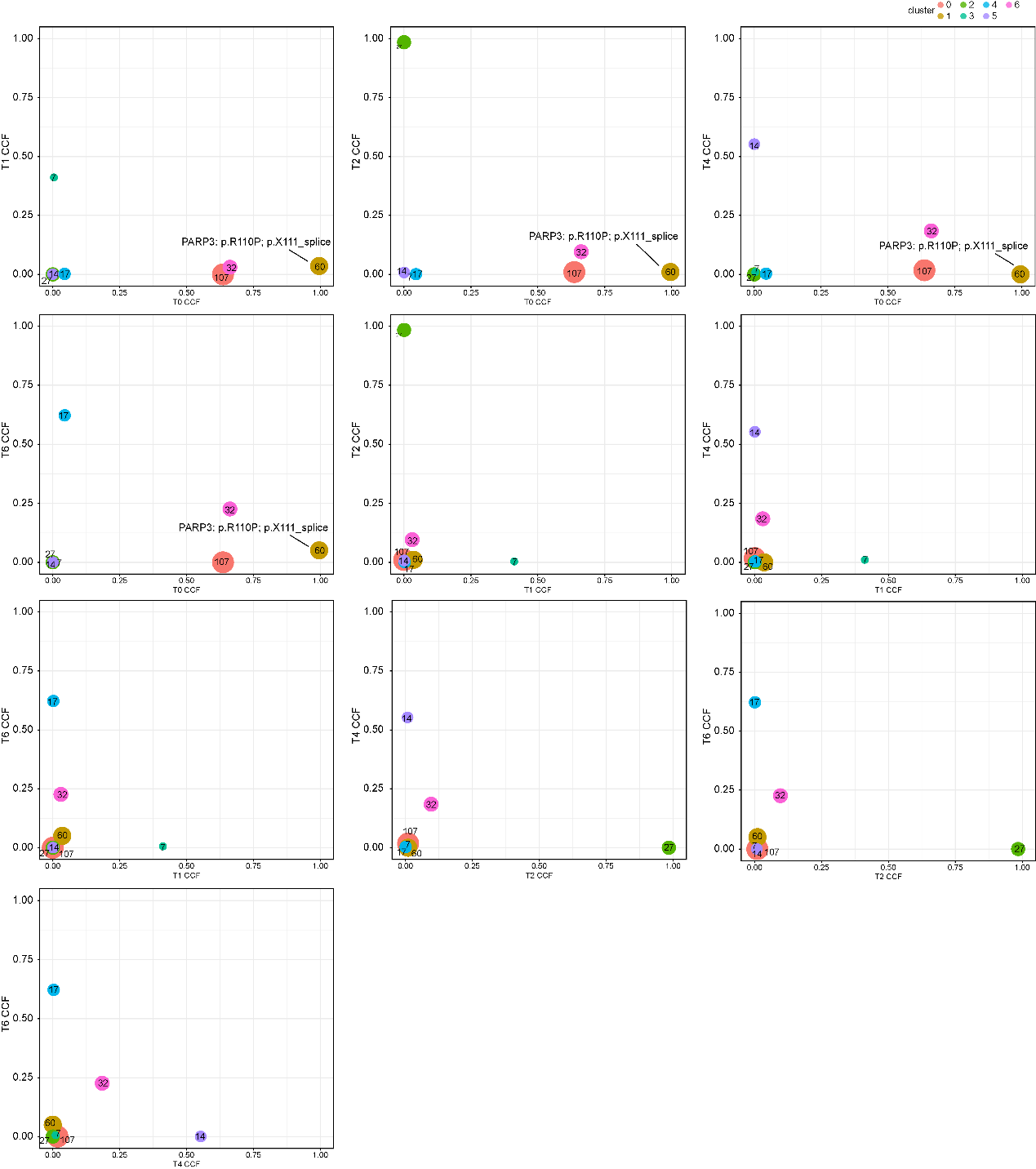
**

**P19
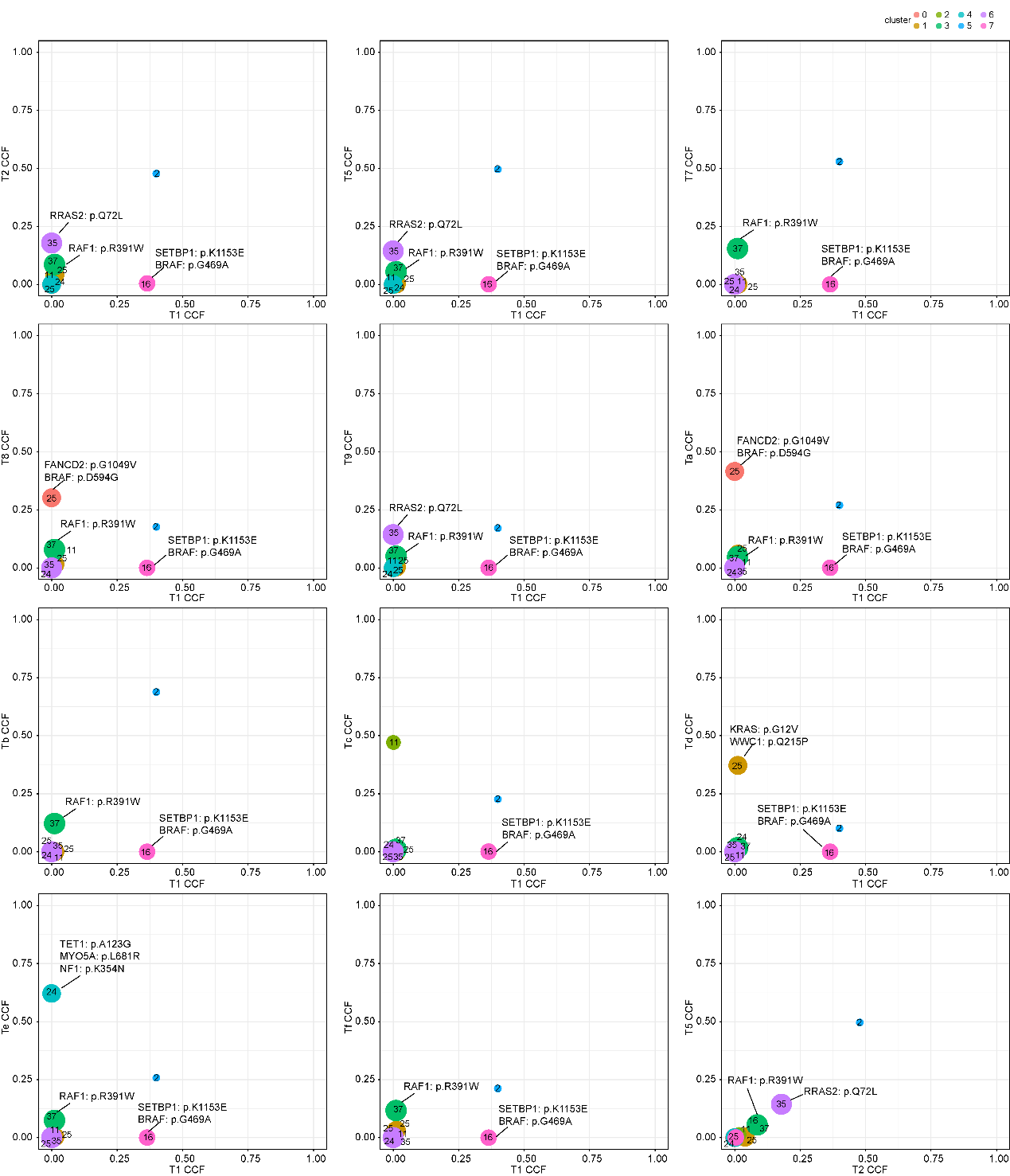

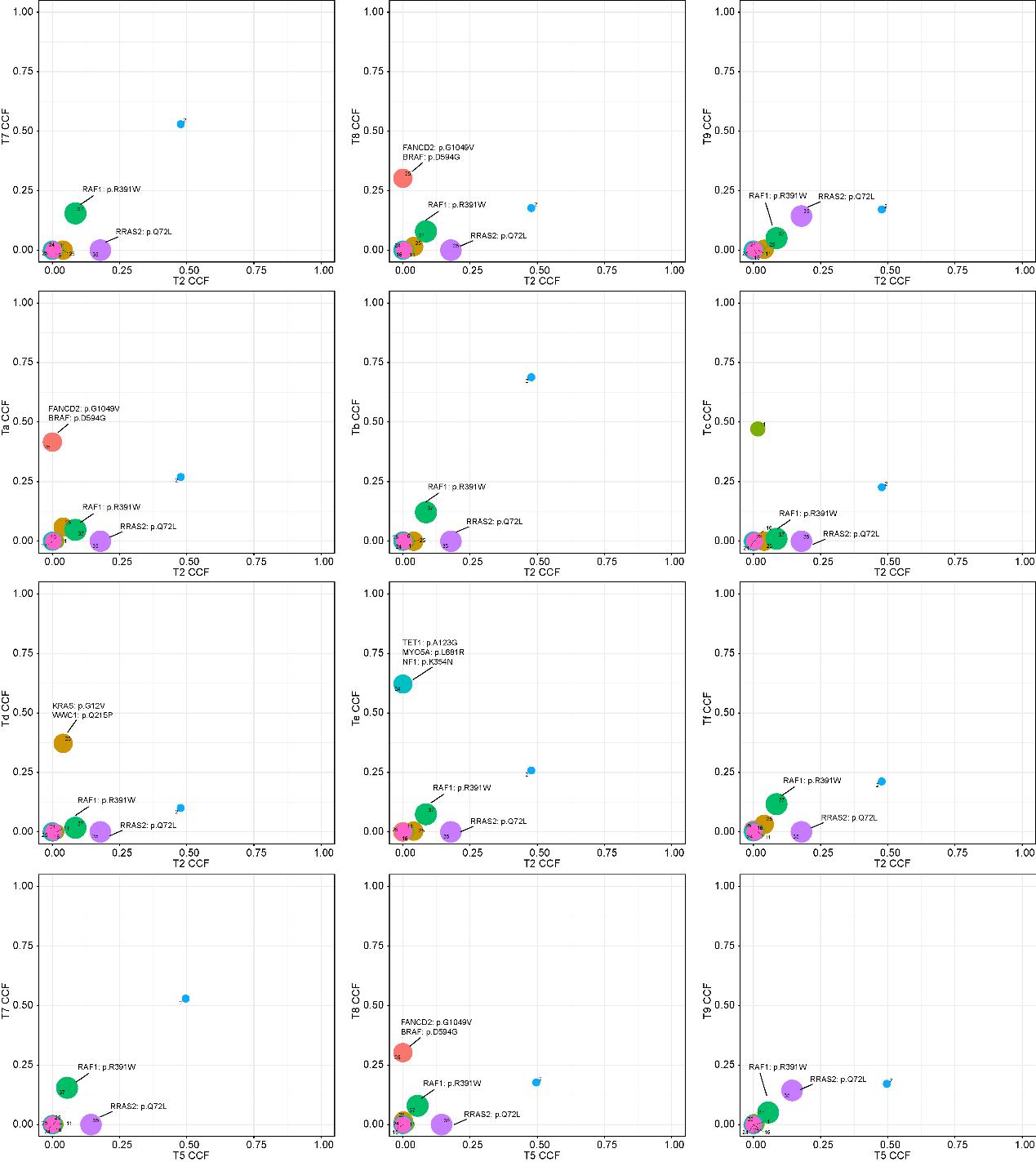

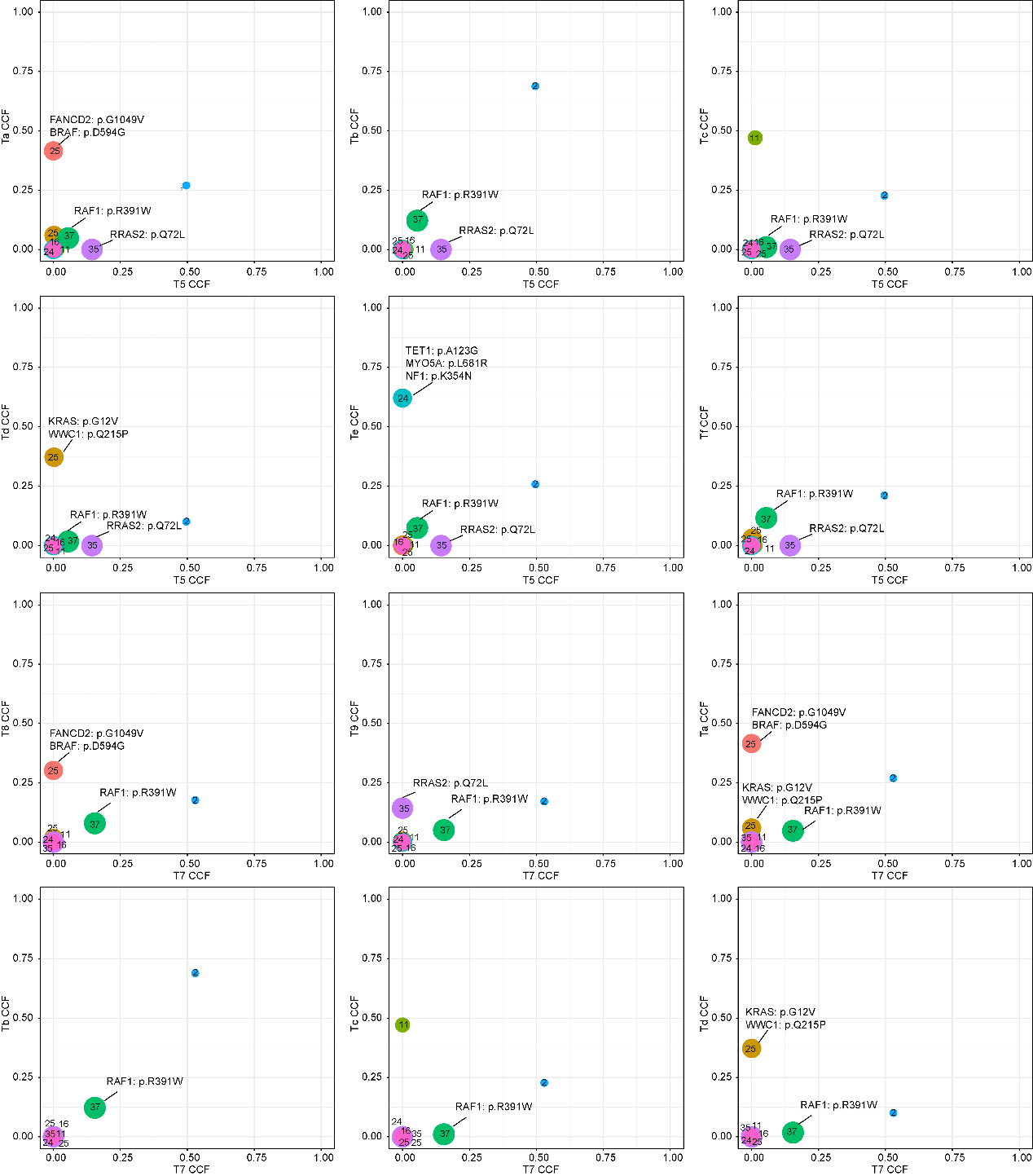

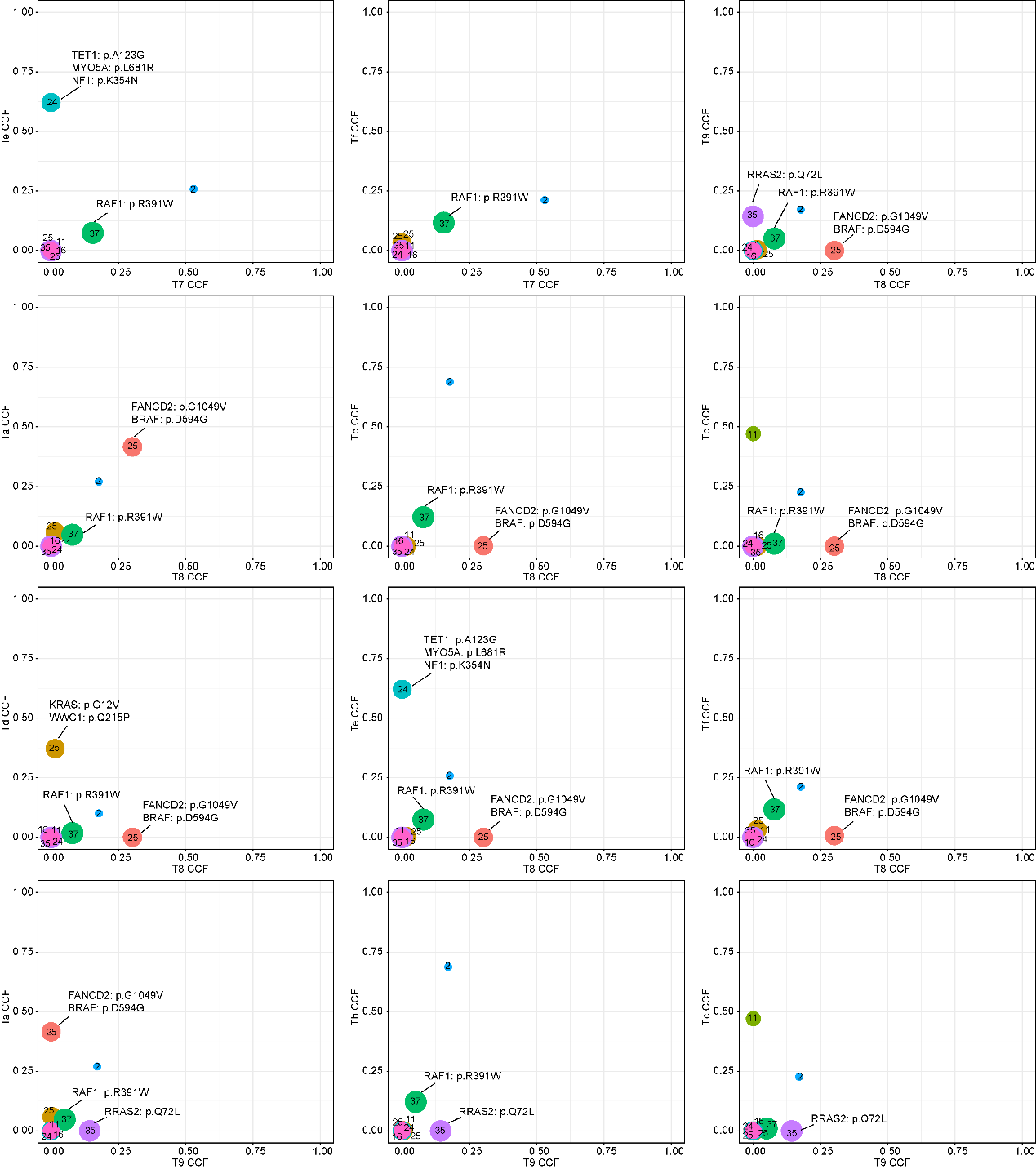

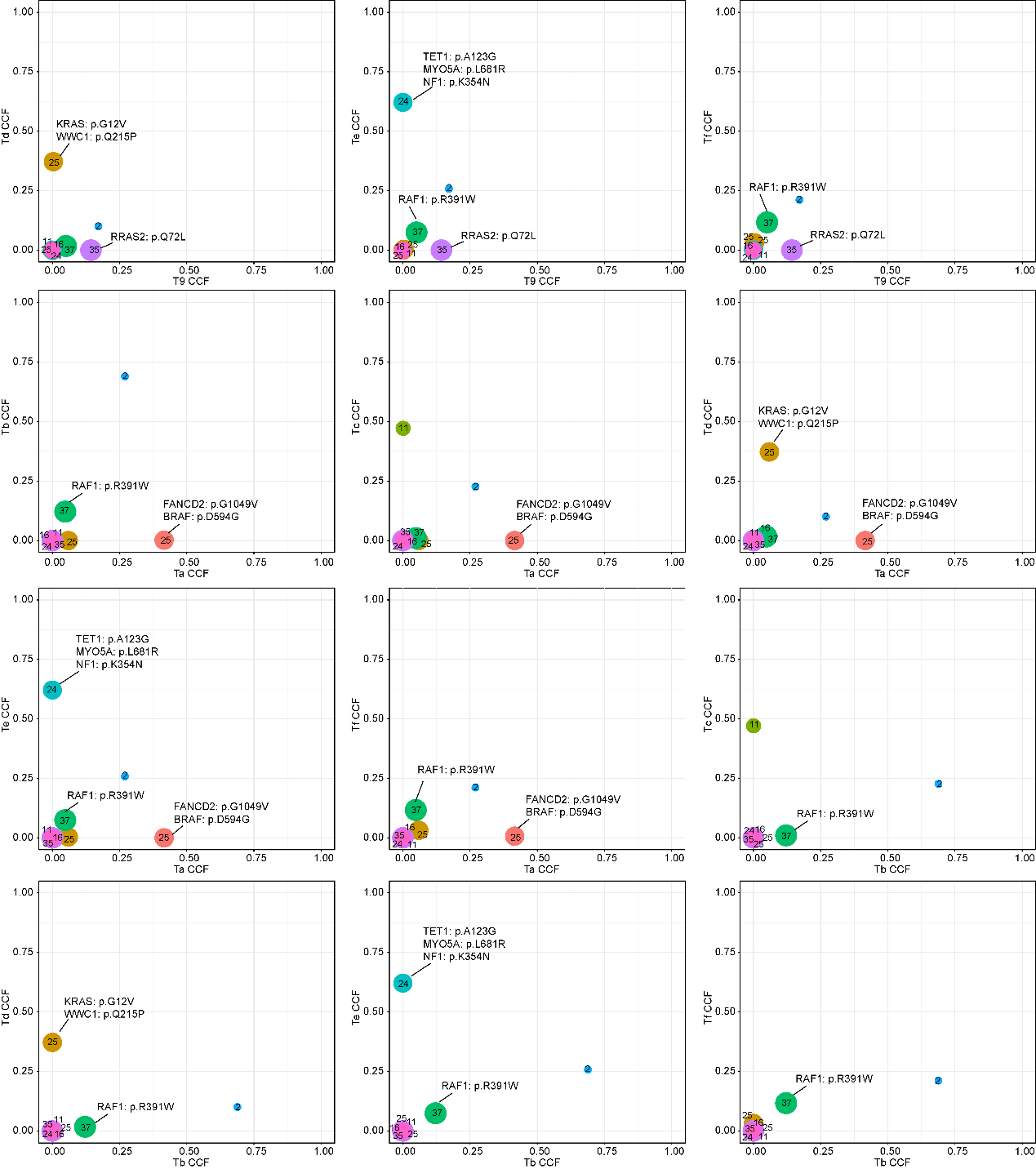

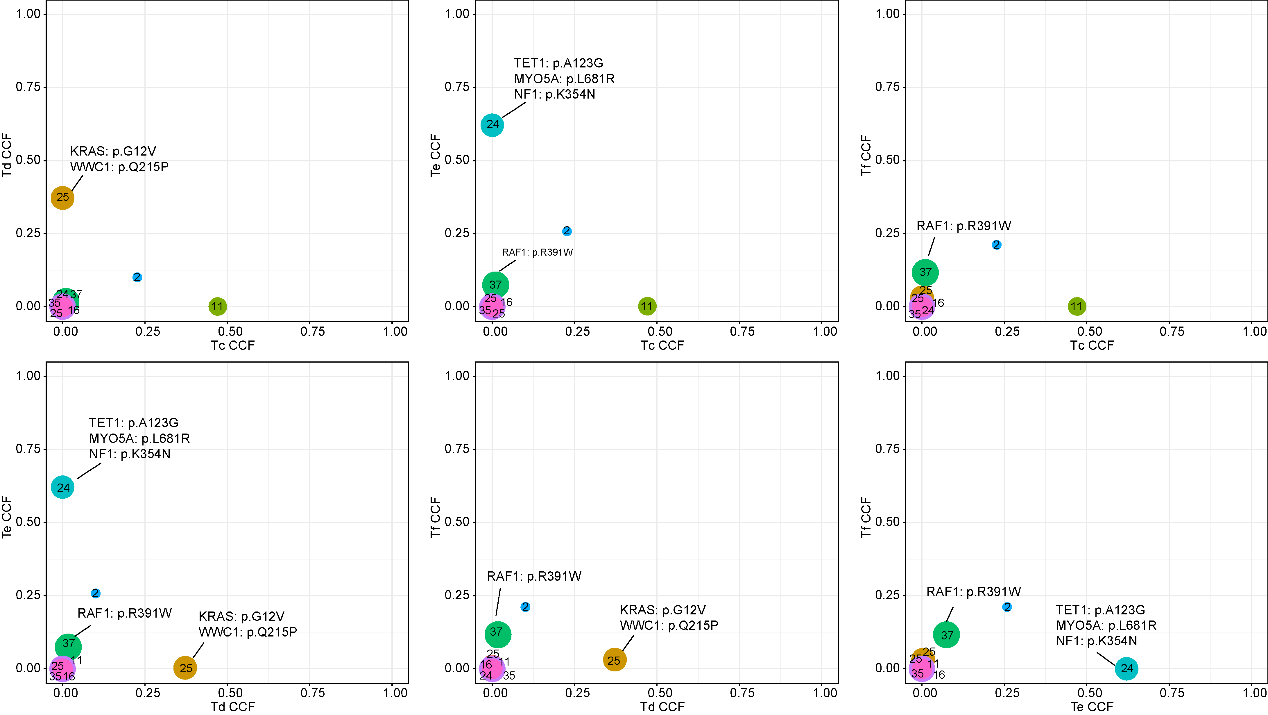
**

**B**

**P04 P06 P11**

**
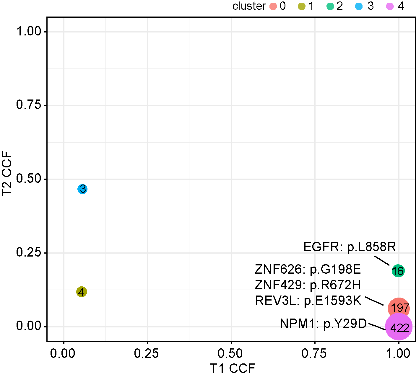

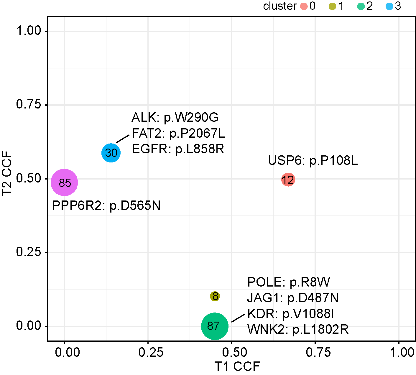

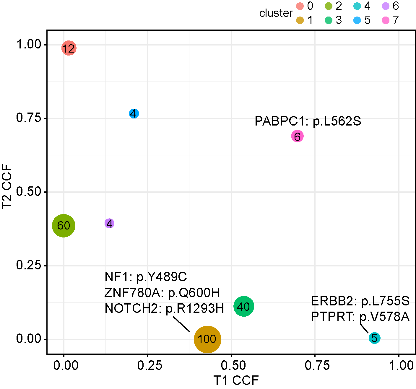
**

**P15**

**
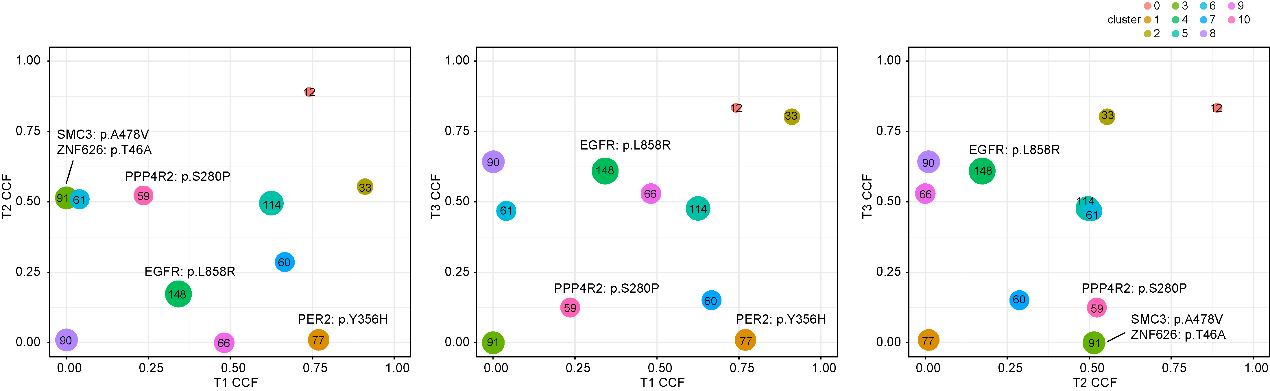
**

**P16**
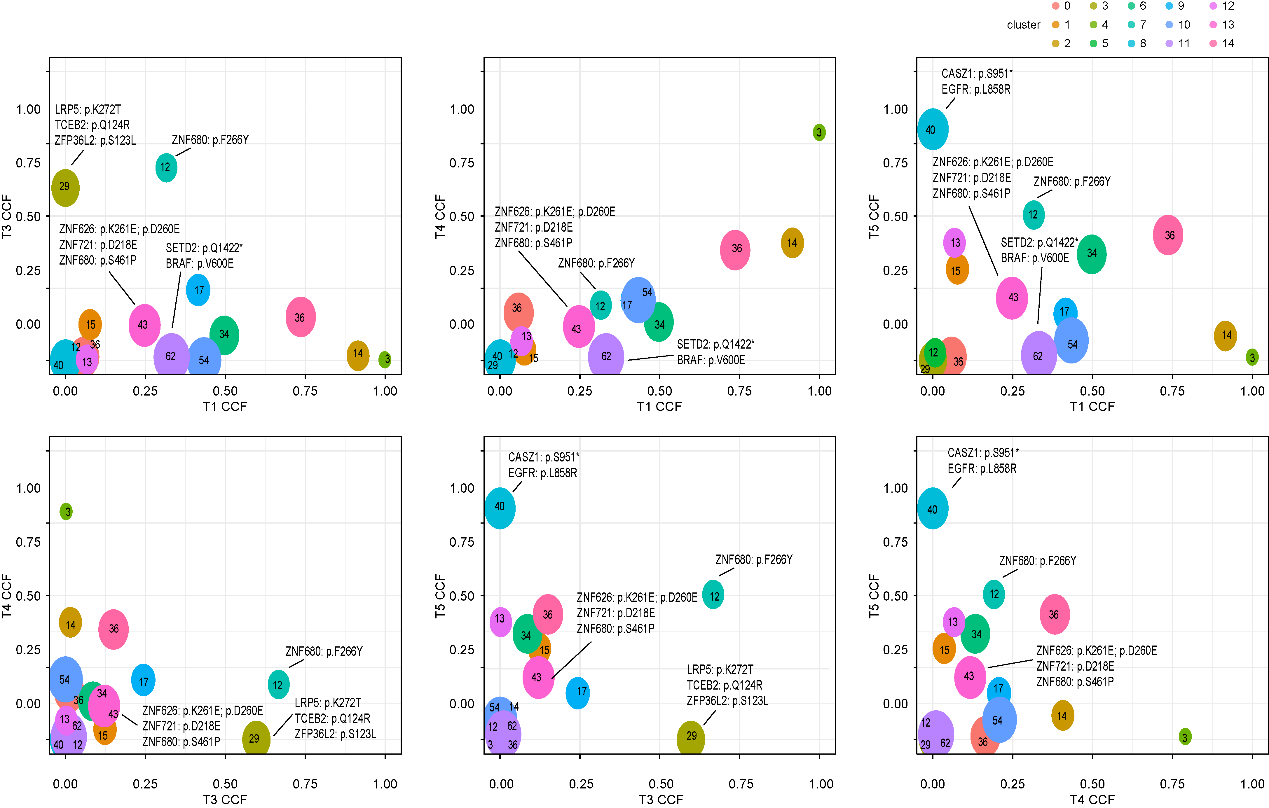


**Supplementary Figure 5.** The clonal relationship between ground-glass nodules (GGNs) in each patient. (A) Patients without shared clonal driver mutations (N=14). (B) Patients with shared clonal driver mutations (N=5).


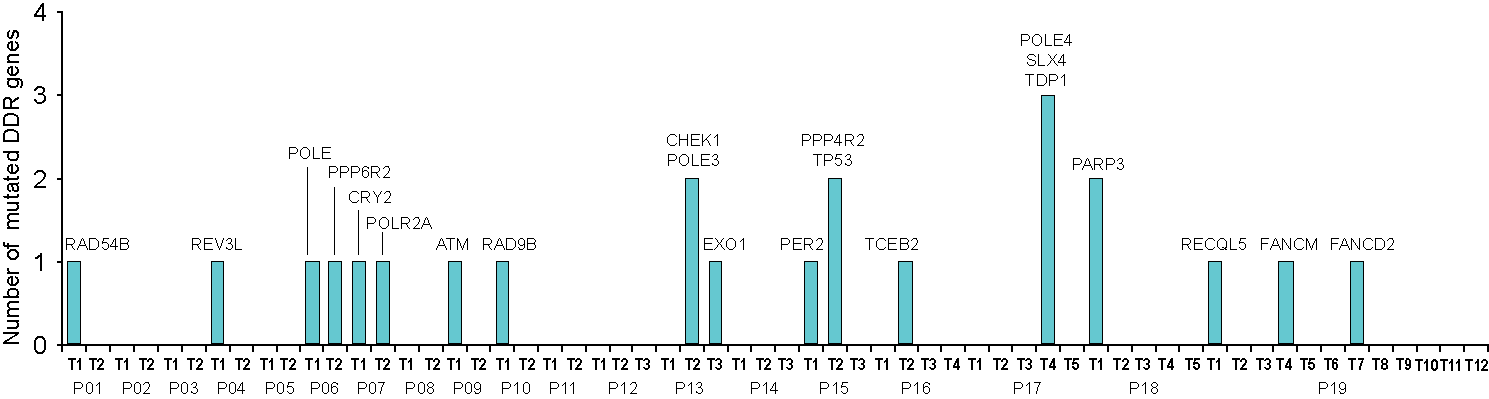


**Supplementary Figure 6.** Mutated DNA damage repair (DDR) genes in each ground-glass nodule (GGN).
